# Characterization of HSP90 isoforms in transformed bovine leukocytes infected with *Theileria annulata*

**DOI:** 10.1111/cmi.12669

**Published:** 2016-10-20

**Authors:** Jane H. Kinnaird, Meetali Singh, Victoria Gillan, William Weir, Ewen D. D. Calder, Isabel Hostettler, Utpal Tatu, Eileen Devaney, Brian R. Shiels

**Affiliations:** ^1^ Institute of Biodiversity Animal Health and Comparative Medicine, College of Medical, Veterinary and Life SciencesUniversity of Glasgow, Garscube CampusBearsden RoadGlasgowG61 1QHUK; ^2^ Department of BiochemistryIndian Institute of ScienceBangalore560 012India; ^3^ Institute for Parasitology, Vetsuisse FacultyUniversity of BernBernSwitzerland

## Abstract

HSP90 chaperones are essential regulators of cellular function, as they ensure the appropriate conformation of multiple key client proteins. Four HSP90 isoforms were identified in the protozoan parasite *Theileria annulata*. Partial characterization was undertaken for three and localization confirmed for cytoplasmic (TA12105), endoplasmic reticulum (TA06470), and apicoplast (TA10720) forms. ATPase activity and binding to the HSP90 inhibitor geldanamycin were demonstrated for recombinant TA12105, and all three native forms could be isolated to varying extents by binding to geldanamycin beads. Because it is essential, HSP90 is considered a potential therapeutic drug target. Resistance to the only specific Theileriacidal drug is increasing, and one challenge for design of drugs that target the parasite is to limit the effect on the host. An *in vitro* cell culture system that allows comparison between uninfected bovine cells and the T. annulata‐infected counterpart was utilized to test the effects of geldanamycin and the derivative 17‐AAG. T. annulata‐infected cells had greater tolerance to geldanamycin than uninfected cells yet exhibited significantly more sensitivity to 17‐AAG. These findings suggest that parasite HSP90 isoform(s) can alter the drug sensitivity of infected host cells and that members of the *Theileria* HSP90 family are potential targets worthy of further investigation.

## 1 INTRODUCTION

Chaperone proteins are fundamental to the enablement of conformational changes in a wide range of cellular effector (client) proteins, especially under conditions of stress (Morimoto, 1998; Kennedy, Jäger, Mosser, & Samali, 2014). Conformational change can result in stabilization, degradation, or activation of client proteins. HSP90 chaperones, however, are particularly known for their tendency to interact with specific proteins involved in essential cellular regulation under non‐stress conditions: client proteins include nuclear hormone receptors, kinases, and transcription factors (Pearl & Prodromou, 2006). HSP90s are essential in eukaryotes, and because of their involvement in so many cellular processes, including proliferation and metastasis, they are the subject of extensive investigation as therapeutic targets for many types of cancer.

*Theileria annulata* is a tick‐transmitted apicomplexan parasite of bovids and is the causative agent of tropical theileriosis. This disease is prevalent from Southern Europe to the Middle East extending into the Far East. Infected cattle have a high mortality as a result of infection, and in cattle that recover a carrier state may develop resulting in significant losses in productivity. Increasingly, the disease is being identified in Mediterranean countries such as Italy, Spain, Portugal, and Greece, possibly due to increased prevalence of the tick vector as a result of global warming. T. annulata undergoes several stage differentiation events during its life cycle that require adaptation to different cellular environments: a sexual cycle occurs in the tick vector, followed by generation of the sporozoite stage in the salivary glands; invasion of the bovine leukocyte by the sporozoite, followed by differentiation to a multinucleate intracellular macroschizont; a switch from the macroschizont stage to production of uni‐nucleate merozoites; invasion of erythrocytes and development into piroplasms, a life cycle stage that may represent gametocytes that are transmitted to the tick vector on feeding (reviewed in (Dobbelaere & Heussler, 1999)). Infection of the leukocyte is accompanied by modulation of the host cell environment, as demonstrated by the radical transformation in phenotype upon establishment of the macroschizont (Baylis, Megson, & Hall, 1995; Baumgartner et al*.*, 2000; Kinnaird et al*.*, 2013). Host cell transformation generates the capacity for unlimited division and development of significant metastatic potential, both of which are associated with the pathogenesis of acute disease. Given the known role of HSP90 in carcinogenesis, host and/or parasite isoforms of HSP90 chaperones may be required for maintenance of the transformed phenotype of the infected leukocyte.

Studies on other closely related Apicomplexans, e.g., *Plasmodium falciparum*, as well as the coccidians *Toxoplasma gondii and Eimeria tenella,* have demonstrated a role for Hsp90 in progression of the life cycle. For example, exposure to the specific Hsp90 inhibitor, geldanamycin (GA) has been shown to disrupt the transition from rings to trophozoites in *P. falciparum* (Banumathy, Singh, Pavithra, & Tatu, 2003); in *Eimeria tenella,* treatment of sporozoites with GA inhibited invasion and development to the schizont (Péroval, Péry, & Labbé, 2006); and for *Toxoplasma*, GA can inhibit the switch from tachyzoite to bradyzoite (Echeverria et al*.*, 2005). Thus, HSP90 plays an important role in enabling stage differentiation events in this parasite group.

One known substrate or client protein of HSP90 is a kinase (IKK) involved in degradation of the inhibitor of the transcription factor NF‐κB. Inhibitor degradation allows translocation of NF‐κB to the nucleus leading to transcriptional activation of numerous target genes, particularly those involved in the inflammatory response and protection against apoptosis. This process is known to occur in *Theileria*‐infected cells (reviewed in (Dobbelaere & Kuenzi, 2004)) where IKK signalosomes, involved in signal transduction of NF‐κB activation, were identified on the surface of the macroschizont (Heussler et al., 2002). However, unusually, no bovine HSP90 was detected within these complexes (Hermann & Dobbelaere, 2006), raising the question as to how functionally competent signalosomes are formed and whether a divergent parasite derived isoform might be involved.

One form of HSP90, encoded by gene *TA12105*, was partially characterized in the closely related parasite, *Theileria parva*, by Gerhards et al. (Gerhards et al., 1994). Using a specific antibody generated against 88 aa at the C‐terminus, they demonstrated that it was localized to the macroschizont cytoplasm, a location typical of the classical form of HSP90. In a more recent study (Mohammed, Bakheit, Ernst, Ahmed, & Seitzer, 2013), the sequence properties of two other HSP90 type proteins, TA06470 (115.6 kDa) and TA10720 (104.2 kDa), were briefly described but no putative function or location was highlighted. The work presented here extends these preliminary analyses for three T. annulata HSPs (TA12105, TA06470, and TA10720) and describes their subcellular localization within macroschizont‐infected cells, binding affinities for GA and expression across different life cycle stages.

A primary aim of this study was to address the potential role of HSP90 isoforms in parasite dependent establishment and modulation of infected host cell phenotype. Moreover, as instances of infections resistant to the theileriacidal drug, buparvaquone, are increasingly recorded in the field (Mhadhbi et al., 2010; Sharifiyazdi, Namazi, Oryan, Shahriari, & Razavi, 2012; Mhadhbi et al., 2015), there is a requirement for development of additional drugs targeted against the parasite that have minimal impact on the host animal. The availability of a wide range of tailored HSP90 inhibitors, together with structural divergence of the apicomplexan HSP90 isoforms from those of the host, highlights their potential as drug targets. The results of this study indicate that there is potential merit in this approach.

## 2 RESULTS

### 2.1 The HSP90 isoforms encoded in the T. annulata genome

**Figure 1:**
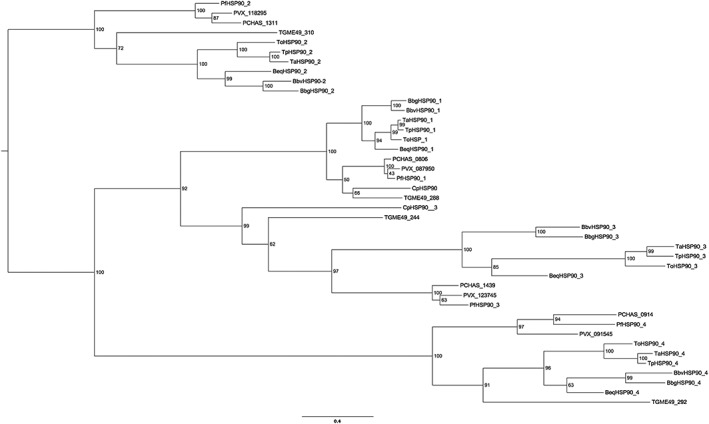
Maximum likelihood tree grouping *Theileria*, *Babesia*, and *Plasmodium* HSP90 variants. Accession numbers for the species used in this analysis are given in Supplementary Table 1B: Species prefixes are Tp, T. parva; To, T. orientalis; Pf, *P. falciparum*; BBov, *B. bovis*; BBig, *B. bigemina*; Beq, *B. equi*; PVX, P. vivax; PCHAS, *P. chabaudi*; TG, *T. gondii*; and Cp, C. parvum. The suffix HSP_1 represents the group containing TA12105. HSP90_2 represents the group containing TA10720. HSP90_3 represents the group containing TA06470. HSP90_4 represents the group containing TA06845

The T. annulata genome sequence (http://www.genedb.org) was analyzed by BLAST for similarities to the mammalian HSP90 protein sequence. Four HSP90‐like proteins were identified: TA12105, the classical, cytoplasmic form of HSP90 (predicted molecular mass of 83.8 kDa), TA06470 (115.6 kDa), TA10720 (104.2 kDa), and TA06845 (83.0 kDa) (summarized in Supplementary Table 1A). The sequence of each of these isoforms was used to identify the most closely related variants in the nearest (*Babesia* and *Plasmodium*) and more distant (*Toxoplasma* and *Cryptosporidium*) genera by BLAST searches of the apicomplexan database at NCBI. A selection was chosen to determine their phylogenetic relationship, and this is presented as a maximum likelihood tree (Figure 1). Four clusters are evident, and, overall, the tree is very stable with good bootstrap support throughout. Each of the four T. annulata isoforms appears in a different cluster, each of which represents an orthologous group. This indicates that four HSP90 variants existed in the common ancestor of the modern apicomplexan genera represented in the analysis. As might be expected, the *Theileria/Babesia* sequences cluster closely for each of the HSP90 types. The *B. equi* sequence is sometimes slightly closer to the *Theileria* species (*n* = 2) and sometimes closer to the other *Babesias* (*n* = 2).

Further characterization of the *Theileria* member of each of these orthologous groups was then undertaken, although, for TA06845, this was limited to noting a predicted amino acid sequence similarity (63%) to the mammalian mitochondrial chaperone, Trap, using T. annulata GeneDB.

### 2.2 TA12105

**Figure 2:**
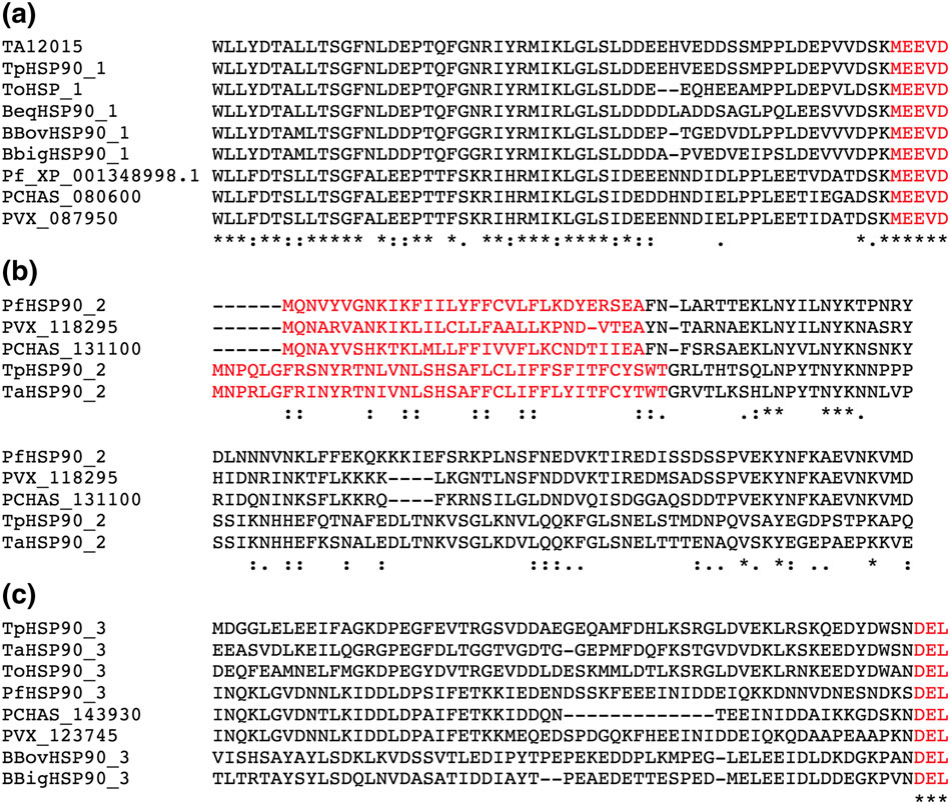
Protein sequence alignment of the defining features of each of the apicomplexan HSP90 isoforms indicating the overall similarity and motif relationship between the orthologues; Ta, *Theileria annulata*; Tp, *Theileria parva*; To, *Theileria orientalis*; Pf, *Plasmodium falciparum*; PVX, *Plasmodium vivax*; PCHAS, *Plasmodium chaubaudi*; Bbov, *Babesia bovis*; Bbig, *Babesia bigemina*. a. Comparison of C‐termini sequences from HSP90‐like proteins in group 1 (cytoplasmic isoform) across *Theileria*, *Babesia*, and *Plasmodium* species indicating conservation of the motif, MEEVD, characteristic of cytoplasmic forms of HSP90. b. Comparison of N‐terminal sequences from putative apicoplast HSP90 proteins (Group 2) from *Theileria* and *Plasmodium* species. The apicoplast targeting sequence is rich in K and N downstream of predicted signal peptide (red). *Babesia* sp. while predicted to be targeted to the apicoplast is not included in the alignment due to the level of divergence (Supplementary Figure 2). c. Comparison of C‐termini sequences from HSP90‐like proteins in group 3 (endoplasmic reticulum isoform) across *Theileria*, *Babesia*, and *Plasmodium* species indicating conservation of an endoplasmic reticulum retention signal sequence. Accession numbers are given in Supplementary Table 1B

The TA12105 gene encodes a predicted protein of 721 amino acids with a molecular mass of 83.8 kDa, possessing 66% identity and 81% similarity to mammalian HSP90 alpha. This HSP90 isoform was first identified by Gerhards et al. (Gerhards et al., 1994). TA12105 contains a conserved C‐terminal MEEVD motif known to be involved in binding of TPR domain containing co‐chaperones such as HOP (Chen, Sullivan, Toft, & Smith, 1998). This motif is typical of all cytoplasmic forms of HSP90 and is absolutely conserved in the other members of the Apicomplexa examined (Figure 2a). As can be seen, the remainder of the C‐terminus is reasonably well conserved across the different species, and this is typical of the whole protein (Supplementary Figure 2).

### 2.3 TA10720

*Theileria annulata* HSPs, TA10720 and TA06470 described below, have not been previously characterized, apart from preliminary sequence data presented by Mohammed et al. (Mohammed et al., 2013). The TA10720 gene predicts a protein of 104.2 kDa and 913 aa, somewhat larger than the cytoplasmic form of HSP90 described above. A potential signal peptide is present at the N‐terminus with an overlapping transmembrane domain, and the protein is predicted to be targeted to the apicoplast. An apicoplast targeting sequence is not well defined, but it has been studied in *P. falciparum* (Foth et al., 2003) where it is rich in K and N residues downstream of a signal peptide, as are the *Theileria* orthologues (Figure 2b). The putative *P. falciparum* orthologue (927 aa) which groups as expected in the phylogenetic tree (Figure 1) is a similar size to the predicted T. annulata protein (913 aa). However, the *B. bovis* and *B. bigemia* orthologues are significantly smaller (795 and 798 aa) (Supplementary Figure 3). Only five of the selected sequences that map to this orthologue group (as shown in the alignment) met the criteria of Foth et al. (Foth et al., 2003) for either a signal peptide or an apicoplast targeting sequence predicted by the PlasmoAP tool at PlasmoDB. However by SignalP3.0 analysis, all putative orthologues contain an N‐terminal signal peptide necessary for transport. Further analysis using recently developed software, ApicoAP (Cilingir, Broschat, & Lau, 2012), designed to address the difficulties in reliably identifying apicoplast targets sequences from Apicomplexans other than *Plasmodium* sp, predicted all the group 2 orthologues analyzed to have an apicoplast targeting sequence within the 80 aa downstream of the signal peptide. This is based on the identification of a region downstream of a signal peptide which is depleted for acidic (D,E) but enriched in polar, hydrophilic, or basic residues (H,K,R,N,Q,S,P,Y). Notably, the *B. bovis* orthologue described here was identified as likely to be targeted to the apicoplast in the analysis that tested the prediction software (Cilingir et al., 2012). Although our analyses (Figures 1 and 2B and Supplementary Figure 3) clearly indicate the relationship between members within group 2 and predict their localization to the apicoplast, the multiple alignment over the first 80 or so amino acids is the region that shows least similarity for *Babesia* sp with the *Theileria* and *Plasmodium* orthologues (Supplementary Figure 3): thus, the *Babesia* sp. are not shown in the short alignment presented in Figure 2b. It is likely that there is considerable divergence in the apicoplast targeting sequence and possibly also in the mechanism of translocation within the Apicomplexa.

### 2.4 TA06470

The gene TA06470 encodes an HSP90‐related protein of 115.6 kDa and 988 aa. As in TA10720, there is a predicted signal peptide, but in addition, there is a conserved endoplasmic retention sequence at the C‐terminus (Chang, Erwin, & Lee,, 1989) (Figure 2c), suggesting it functions as an ER chaperone in a similar fashion to mammalian GRP94. Generally, over the whole protein, there is good conservation (Supplementary Figure 4) within the Apicomplexa. The closest orthologues amongst the Apicomplexa found by BLAST searching are the predicted endoplasmins (average MW 94 kDa) from *Plasmodium* spp. and the *Babesia* HSP90s (Supplementary Table 1B). Two B. orientalis HSP90s, including an orthologue of TA06470, were recently the subject of a detailed computational structural analysis (Khan et al., 2014). Alignment of the selection of Apicomplexan HSP90s within this group (Supplementary Figure 4) indicates variably positioned insertions downstream of the ATP binding domain suggesting divergent functions and/or different client proteins.

### 2.5 Expression profiles across T. annulata life cycle stages

Expression profiles of the three *Theileria* HSP90 genes (TA12015, TA10720, and TA06470) across the different life cycle stages associated with bovine infection were generated from a published T. annulata microarray dataset (Pieszko, Weir, Goodhead, Kinnaird, & Shiels, 2015) that represents expression levels for each parasite gene in the genome at the sporozoite, macroschizont, merozoite, and piroplasm stage. There was no significant difference in RNA expression levels among the stages, indicating that these genes are likely to be constitutively expressed throughout the life cycle (Supplementary Figure 5). In addition, no significant increase in expression was observed when merogony was induced by culture at 41°C, with time points examined representing the intermediate phase (Day 4) and Days 7 and 9 where merogony was shown to occur. However by immunoblot analysis, compared to the level of constitutively expressed β‐tubulin, a small increase (<2‐fold) in levels of cytosolic TA12105 protein was observed in *Theileria*‐infected cells following a short (2 hr) heat shock at 42°C indicating that expression of TA12105 may be influenced by a stress response. A small reduction was observed after a recovery period at 37°C, suggesting that TA12105 is relatively stable with a slow turnover rate (data not shown).

### 2.6 T. annulata HSP90 proteins show different cellular localizations

**Figure 3:**
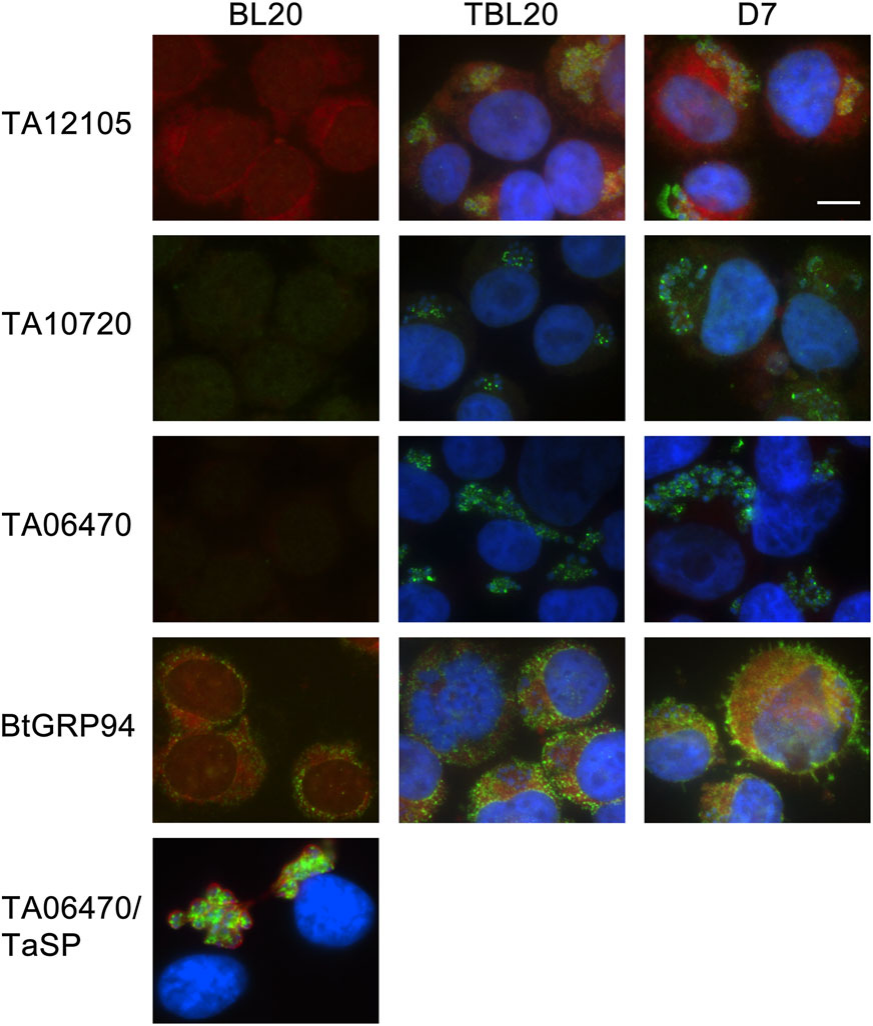
Comparative reactivity by immunofluorescence of anti‐sera to parasite HSP90 isoforms and to bovine GRP94. BL20 is an uninfected bovine cell line used as a control, TBL20 is BL20 infected with *T. annulata* and D7, an *in vivo* derived cloned *T. annulata* infected cell line. Host and parasite nuclei were co‐stained with DAPI (blue) and primary antibody reactivity detected with Alexa 488 (green). TA12105, anti‐peptide anti‐serum to TA12105, the cytoplasmic form of HSP90; TA10720, anti‐serum to TA10720, the apicoplast form of HSP90; TA06470, anti‐serum to TA06470, the endoplasmic reticulum HSP90; BtGRP94, a commercial anti‐serum against mammalian GRP94, which resides in the lumen of the ER; TA06470/TaSP, *T. annulata* TaC12 cells co‐stained with anti‐TA06470 and anti‐TaSP which reacts with the macroschizont membrane (red). Bar is 10 μm

**Figure 4:**
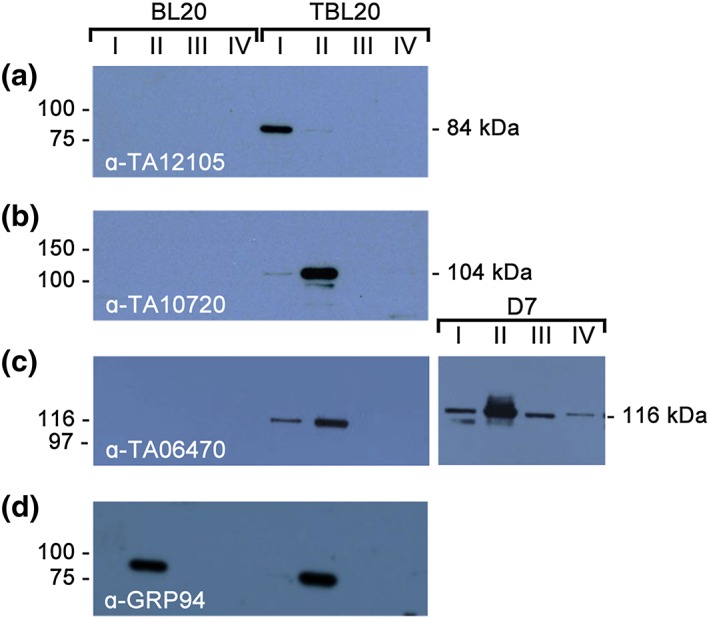
Comparative Western blot analysis of antibody reactivity to *Theileria* HSP90 variants and bovine host GRP94 in subcellular fractions prepared from BL20 (uninfected) and TBL20 (*Theileria*‐infected) cells. Extracts were prepared using the S‐PEK cell fractionation kit (Pierce). Fraction I, enriched for cytoplasmic proteins; Fraction II, enriched in membranes and organelles; Fraction III, enriched for nuclei; and Fraction IV, residual insoluble proteins including intermediate filaments

The specific antibodies to TA12105, TA10720, and TA06470 were used to determine the localization of each of the three T. annulata HSP90 proteins within the macroschizont by immunofluorescence (IFAT) and subcellular fractionation. IFAT using an anti‐peptide antibody confirmed the predicted cytoplasmic localization of TA12015 in TBL20 and cloned cell line, D7, where the parental line derived from an infected animal (Figure 3). Some concentration of the antigen around the macroschizont nuclei was also observed. No other cellular localization was detected. When infected cells were fractionated into four different cellular compartments, a polypeptide of 83 kDa was detected predominantly in Fraction I, the cytoplasmic fraction (Figure 4A) with only a small amount in Fraction II, which is enriched for membranes and organelles. As TA12105 appears to be quite highly expressed, this most likely represents carry over during fractionation or alternatively, a proportion may be associated with internal membranes.

Reactivity against a polypeptide of 83 kDa in the piroplasm stage was not detected by immunoblot (data not shown); rather, three predominant reactive polypeptides of 75, 50, and 45 kDa were observed. Similarly, Gerhards et al. (Gerhards et al., 1994) did not detect any reactivity in piroplasms using an antibody generated against 663 aa at the C‐terminus, although an RNA species of the correct size was present, in agreement with our microarray expression data for this gene. The results indicate expression of the gene as a polypeptide that subsequently undergoes cleavage in the piroplasm stage or during production of the piroplasm protein extract. An antiserum directed against a different piroplasm protein, a 30–32 kDa surface antigen (Glascodine et al., 1990), showed it to be intact and of the correct molecular mass, indicating protein cleavage/degradation is not a general phenomenon (data not shown). Using the antiserum against TA12105, no reactivity was observed with the uninfected control cell line BL20, either by IFAT or Western blot, demonstrating specificity for infected cells (Figures 3 and 4A).

### 2.7 TA10720

For TA10720, the polyclonal antibody generated against the C‐terminus recognized several clear punctate structures within the macroschizont of each infected cell, most likely to be apicoplast bodies (Figure 3), supporting the predicted location. By Western analysis, the predominant polypeptide of ~110 kDa matches the predicted size of 104 kDa for TA10720 (Figure 4B) and was detected predominantly in Fraction II, which is enriched for membrane and organelles. A small amount is detectable in cytoplasmic Fraction I. No reactivity was detected against the uninfected control cell line BL20.

### 2.8 TA06470

The polyclonal antibody generated to the C‐terminus of TA06470 predominantly recognized an extensive network of membranous‐like strands linking multiple, bright, punctate bodies within the macroschizont of TBL20 and D7 cell lines (Figure 3). By Western analysis of subcellular fractions, a polypeptide of ~120–130 kDa (the predicted MW for TA06470) was detected that is specific to infected cells (Figure 4C). In these studies, TA06470 partitioned predominantly with membranes and organelles (Fraction II) in TBL20 (Figure 4C), but a significant amount was also observed in the cytoplasmic fraction. In the D7 cloned cell line, TA06470 was also associated with the cytoplasmic fraction and smaller amounts in nuclear (Fraction III) and the Fraction (IV) containing residual insoluble material such as intermediate filaments of the cytoskeleton. When the same extracts were probed with a commercial antibody to mammalian GRP94, an HSP90 typically found in the lumen of the endoplasmic reticulum, no signal was detected in the cytoplasmic fractions of either BL20 or TBL20 (Figure 4D). The observation that TA06470 is detected in other cellular compartments by Western blot could indicate a low level of secretion into the host compartment, but no clear evidence for this was found by IFAT on co‐staining with an antibody that demarcates the surface of the macroschizont (anti‐TaSP, Figure 3).

### 2.9 GRP94

In common with recent observations for other HSPs (reviewed in (Tsutsumi & Neckers, 2007; Wong & Jay, 2016)), mammalian GRP94 (also known as GP96), which is typically present in the ER, has been shown to exist in minor quantities on the cell surface of transformed cells but not normal cells (Altmeyer et al., 1996). IFAT using anti‐GRP94 detected a close association with the cell surface/periphery in TBL20, and this pattern was particularly evident in the infected D7 cell line (Figure 3). In addition, GRP94 appears to be significantly more abundant in the two T. annulata infected cell lines compared to uninfected BL20 cells. This observation was supported by examination of a previously published microarray data set that compares host gene expression in BL20 and TBL20 cells (Kinnaird et al., 2013). The data indicated that the bovine GRP94 gene exhibited statistically significant (FDR 0.02) elevation in expression (>1.4‐fold absolute) in TBL20 compared to BL20 and that this increased expression was reversible when TBL20 cells were exposed to the anti‐parasite drug, buparvaquone.

### 2.10 Native HSP90 isoforms from T. annulata‐infected cells show variable binding to geldanamycin

**Figure 5:**
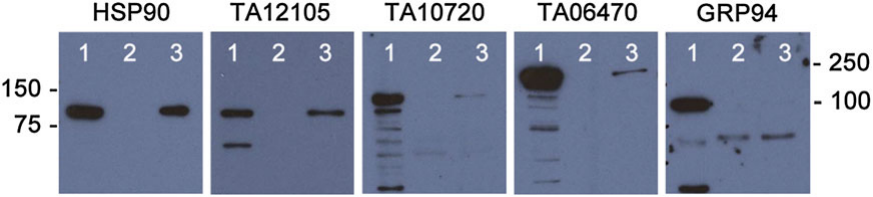
Detection of the geldanamycin binding affinities of HSP90 variants present in detergent extracts of T. annulata infected cells using biotinylated geldanamycin immobilized on streptavidin magnetic beads: Bovine HSP90 (HSP90); T. annulata cytoplasmic HSP90 (TA12105); T. annulata apicoplast HSP90 (TA10720); T. annulata endoplasmic reticulum HSP90 (TA06470); and Bovine GRP94 associated with host endoplasmic reticulum (anti‐mammalian GRP94). Track 1, total detergent extract; track 2, eluate from beads exposed to DMSO alone; track 3, eluate from streptavidin beads pre‐bound with biotin‐GA

The abilities of different HSP90s present in detergent extracts of *Theileria*‐infected cells to bind to the HSP90 inhibitor, geldanamycin (GA), were assessed by capture on GA magnetic beads followed by detection of eluted protein with the specific HSP90 antibodies. Both mammalian and T. annulata (TA12105) cytoplasmic forms of HSP90 were clearly detected in the eluate from GA‐beads indicating that TA12015 also had significant affinity for GA. Estimates of the ratio of total protein reactivity relative to that of eluted protein by densitometry generated a lower ratio for TA12015 (1.5 versus 2.2 for host, Figure 5). Interestingly, both TA10720 (apicoplast) and TA06470 (parasite ER) could bind to a limited degree, as low reactivities were detected in the eluate. Virtually, no host GRP94 was detected in the eluate indicating very limited binding to GA‐beads (Figure 5).

### 2.11 Recombinant TA12105 has ATPase activity

**Figure 6:**
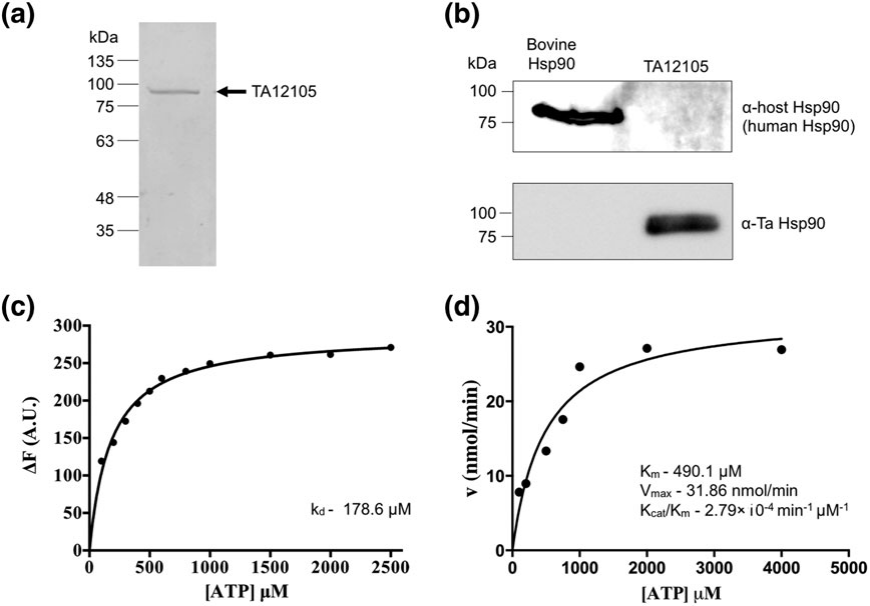
TA12105 (cytoplasmic HSP90) is shown to be a functional ATPase. a. The quality of purified full‐length recombinant TA12015 was assessed following resolution by 10% SDS polyacrylamide gel electrophoresis and Coomassie Blue staining. b. Immunoblotting and detection using the antibody specific to TA12015 compared to an antibody specific for bovine host HSP90. c. Binding affinity of recombinant TA12015 for ATP measured using a fluorescence quenching ATP binding assay; the dissociation constant (kd) was 178.6 μM. d. Analysis of Michaelis–Menten kinetics by ɤ‐P^32^‐ATP hydrolysis

**Figure 7:**
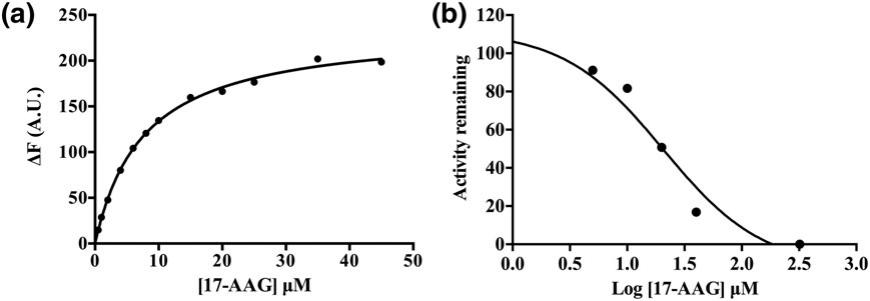
A. Hsp90 inhibitor 17‐AAG can bind to and inhibit the ATPase activity of recombinant TA12105. a. Recombinant TA12105 binding to a classical HSP90 inhibitor, 17‐AAG in a fluorescence‐quenching assay with a Kd of 7.67 μM. b. ATPase activity of recombinant TA12105 inhibited by addition of 17‐AAG in an ɤ‐P32‐ATP hydrolysis assay: the IC_50_ for 17‐AAG was 20.59 μM

To investigate the properties of the cytoplasmic form of T. annulata HSP90, TA12105, compared to those of the bovine orthologue, full‐length recombinant TA12105 was expressed in E. coli as a histidine‐tagged fusion protein and affinity purified (Figure 6a). As in our previous experiments using native protein, the purified fusion protein reacted strongly with the specific peptide antiserum to TA12105 while an antibody specific for bovine HSP90 showed no cross‐reactivity with the recombinant protein by immunoblot analysis (Figure 6b). The binding affinity of recombinant TA12015 for ATP was measured using a fluorescence quenching ATP binding assay and the dissociation constant (K_d_) calculated to be 178.6 μM. It is known that the affinity of recombinant HSP90 for ATP and ATPase activity is low *in vitro* and that *in viv*o, HSP90 activity is highly regulated by the presence of a wide range of co‐factors such as Aha1, CDC37, and p23 that alter the conformation (reviewed in (Prodromou, 2012)). By using a ɤ‐P^32^‐ATP hydrolysis assay, a measurable ATPase activity was obtained for recombinant TA12015 with a Km of 490.1 μM indicating that TA12105 is a functional ATPase (Figure 6d). When binding of HSP90 inhibitor 17‐AAG to recombinant TA12105 was measured by a fluorescence quenching assay, a K_d_ value of 7.67 μM was obtained (Figure 7a). Furthermore, the ATPase activity of recombinant TA12105 could be inhibited by addition of 17‐AAG (Figure 7b) with an IC_50_ calculated as 20.59 μM. These results indicate that the protein encoded by TA12105 has functional properties similar to a classical HSP90 that can be blocked by the inhibitor 17‐AAG.

### 2.12 HSP90 inhibitors differentially affect growth of T. annulata‐infected leukocytes

**Figure 8:**
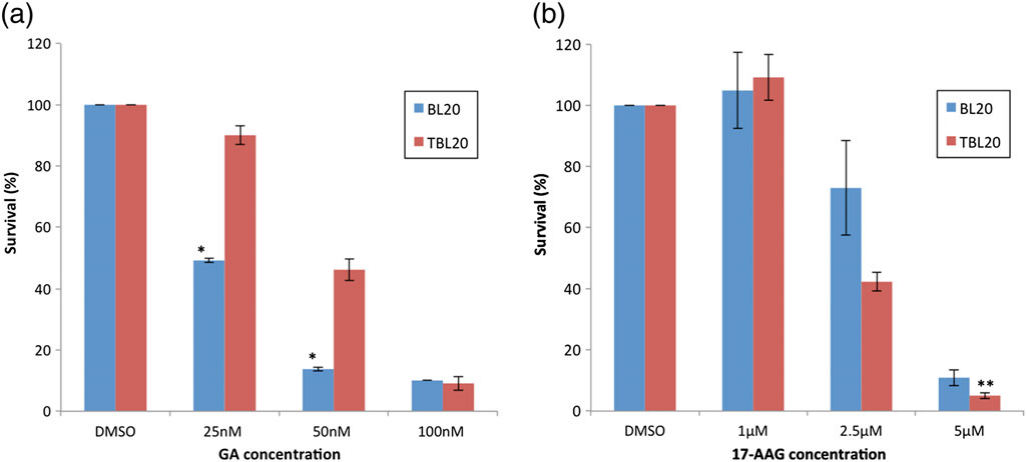
Differential survival of *Theileria* infected relative to uninfected cells when treated with HSP90 inhibitors Geldanamycin and 17‐AAG. Cultures were set up in triplicate at 1.4 × 10^5^ ml^−1^. Geldanamycin and 17‐AAG added at 25, 50, and 100 nM for GA (a) and 1, 2.5, and 5 μM for 17‐AAG (b); control cultures contained DMSO. Results are expressed as percent survival after drug treatment compared to control when counted using Trypan Blue exclusion at 48 hr. Differences in survival between BL20 and TBL20 at 25 nM (*P* = 0.00285) and 50 nM GA (*P* = 0.00503) were statistically significant (*) by Students *t*‐test whereas the slight difference observed at 100 nM GA was not (*P* = 0.547). For 17‐AAG, the detected decrease in survival for TBL20 compared to BL20 was borderline significant at 2.5 μM (*P* = 0.06465) and statistically significant at 5 μM (**, *P* = 0.04868). Whereas at 1 μM 17‐AAG the marginal increase for TBL20 was not significant (*P* = 0.74517). Duplicate experiments gave almost identical results in both cases

Given that all three *Theileria* HSP90s tested were found to bind to GA beads albeit to different extents, experiments were performed to establish if there could be a differential effect of GA on uninfected BL20 cells compared to *Theileria*‐infected TBL20 cells. Cell number and survival were monitored after 48 hr in the presence or absence of drug. When compared to the control cultures exposed to the DMSO vehicle only, BL20 cells were much more sensitive to GA at concentrations that had significantly less effect on parasite‐infected TBL20 (Figure 8a). Thus, at 25 nM GA, 90% of TBL20 cells were viable compared to only 49% in BL20 (*P* = 0.00285, BL20 vs TBL20); at 50 nM, 46% of TBL20 cells were viable but only 14% of BL20 (*P* = 0.00503, BL20 vs TBL20). In contrast, at 100 nM GA, both BL20 and TBL20 were similarly sensitive to GA with only 10% and 9% respectively viable cells, compared to control cultures (Figure 8a). The IC_50_ for BL20 is 24.5 nM and, for TBL20, 48.0 nM.

A similar experiment was carried out using 17‐AAG because the effects of this derivative of geldanamycin could differ to those of GA, particularly *in vivo* (Maroney et al., 2006). For 17‐AAG, an opposite effect to that of GA was observed, as infected TBL20 cells were more sensitive to the drug at 2.5 and 5 μM concentrations than uninfected BL20 (Figure 8b). For BL20, 73% of cells remained viable in 2.5 μM 17‐AAG whereas for TBL20, only 41% were viable compared to the DMSO control cultures (*P* = 0.06465). At 5 μM, while survival was poor in both cell lines, TBL20 remained more sensitive (*P* = 0.04868). About 1 μM 17‐AAG had no significant effect on growth of either BL20 or TBL20 compared to the respective DMSO control (*P* = 0.74517). For TBL20, the IC_50_ was 2.3 μM compared to 3.4 μM for uninfected BL20 cells.

## 3 DISCUSSION

Genes encoding HSP90 isoforms are conserved throughout the Apicomplexa. It is likely that some or all perform functions that are critical for cellular survival and show diversity relative to host isoforms; thus, HSP90 may provide a target for rational design of novel therapeutics against apicomplexan parasites. In addition, either host or parasite HSP90 may play a key role in establishing or maintaining the transformed phenotype of the T. annulata‐infected leukocyte. Four T. annulata proteins encoded by genes belonging to the HSP90 family were aligned with orthologues from other closely related apicomplexan parasites and a phylogenetic tree derived. Each of the four isoforms clustered in one of four different orthologous groups, indicative of a close relationship between the isoforms within the Apicomplexa. However, whilst clearly related with conservation at the amino acid level in certain key domains, such as the S/NKDEL domain at the C‐terminus of endoplasmic reticulum forms, these orthologues also showed regions of considerable divergence. For example, the targeting domain of the otherwise related apicoplast located isoforms showed significant divergence, which may reflect species‐specific differences in mechanism, function, or client proteins during the life cycles. Expression of RNA was constitutive for the three HSP90 genes analyzed, as might be expected for proteins with critical chaperone functions across multiple stages of the life cycle. However, we did not detect full‐length TA06470 protein in piroplasms perhaps suggestive of cellular processing. Alternatively, proteolytic cleavage of this HSP90 isoform may occur during the piroplasm purification procedure from whole blood, where considerable variation can occur between different preparations. Proteolytic cleavage may be the most likely explanation to account for our observations as HSP90 cleavage near to the N‐terminus has been reported to occur in leukaemic cell lines during conditions of oxidative stress (Beck et al., 2012).

By a combination of subcellular fractionation and IFAT, we confirmed localization of TA12105 to the macroschizont cytoplasm, TA10720 to the apicoplast and TA06470 to be highly abundant and very likely located in the parasite endoplasmic reticulum. TA06470 has a reasonably well‐conserved version (S/NDEL) of an ER retention sequence (KDEL) at the C‐terminus (Munro & Pelham, 1987), a feature in common with other HSP90s that have a chaperone function in the ER. Subcellular fractionation studies indicated that small amounts of TA06470 and/or TA10720 might be secreted into the host compartment. There are two reasons for considering this to be possible; in *Plasmodium falciparum*, HSP70 has been detected in the parasitophorous vacuole (Grover, Chaubey, Ranade, & Tatu, 2013), and secondly, in *Theileria* infected cells, the process that results in activation of transcription factor NF‐κB does not appear to require bovine HSP90 nor do IKK signalosome complexes recruited to the surface of the macroschizont and involved in parasite‐dependent activation of NF‐κB appear to contain detectable bovine HSP90 (Hermann & Dobbelaere, 2006). In the mammalian cell, IKK complexes typically contain HSP90 (Chen, Cao, & Goeddel, 2002) to facilitate appropriate protein conformations, thus the following question arises: how does the *Theileria*‐infected cell circumvent this fundamental requirement, and could it involve a parasite derived HSP90 isoform? However, IFAT analysis to co‐detect TA06470 and the macroschizont membrane surface protein, TaSP, did not reveal any evidence of localization on the parasite surface or outside in the host compartment, and a similar result was obtained using anti‐TaSP together with anti‐TA10720 and TA12015 (data not shown). Thus, the formation of IKK signalosomes appears to be independent of host or parasite HSP90, and the presence of a significant amount of TA06470 in the cytoplasmic fraction is most likely to be due to location in the macroschizont cytoplasm. Alternatively, the ER within the macroschizont may be more fragile than that of the host and be more easily subject to mechanical disruption during extraction procedures, because host GRP94 was not detected in the cytoplasmic fraction from either BL20 or TBL20 cells.

Mammalian GRP94 has been reported outside the ER and also in small quantities on the cell surface (Altmeyer et al., 1996), particularly in tumor cells (reviewed in (Marzec, Eletto, & Argon, 2012)) where it is associated with stimulation of innate and adaptive anti‐tumor immune responses (reviewed in (Luo and Lee, 2013)). In addition, upregulation of GRP94 in cancer cells has been proposed to occur in response to ER stress and is considered to be pro‐oncogenic and promote tumor cell migration (Zheng et al., 2008; Rachidi, Sun, & Li, 2015). Therefore, the observation that GRP94 detected by IFAT is significantly upregulated in *Theileria‐*infected cells, with evidence (for the *ex‐vivo* derived D7 cell line in particular) of sequestration at the periphery of the host cell, may be functionally relevant (Figure 3). One possible reason for upregulation of GRP94 gene expression lies in the extensive modification of gene expression that the macroschizont stage of T. annulata imposes on the host cell (Kinnaird et al., 2013). This includes upregulation of many genes encoding cellular proteins such as exported MMPs, chemokines, signaling proteins, and transcription factors which presumably would demand a higher level of chaperoning through the ER. Moreover, the recent link between GRP94 and transport of filamentous actin and integrin to the cell periphery and regulation of cellular migration may also be relevant (Ghosh et al., 2016). Thus, it is possible that modulation of host GRP94 plays an important role in promoting the phenotype of the transformed cell in terms of dissemination throughout and interaction with the host lymphoid system.

Both cytoplasmic forms of bovine and *Theileria* HSP90 clearly bound to GA‐beads with similar affinity. The slightly lower ratio of total protein to eluted protein obtained for TA12105 compared to that for the bovine host isoform may indicate that the parasite protein has higher affinity for GA as observed for the *P. falciparum* orthologue (Pallavi et al., 2010). In contrast to TA12105, parasite ER HSP90, TA06470, and apicoplast HSP90, TA10720 showed limited affinity for GA relative to the cytoplasmic form. Previous studies have demonstrated different sensitivities to inhibitors between *P. falciparum* cytoplasmic HSP90 and mammalian Hsp90 (Pallavi et al., 2010) using recombinant HSP90 and also when expressed in yeast (Wider, Péli‐Gulli, Briand, Tatu, & Picard, 2009). Mammalian GRP94 has been reported to bind to GA (Immormino et al., 2009), but using TBL20 extracts, we detected only a very small amount of binding to GA beads. This raises the possibility that GA affinity for or access to GRP94 might be different between infected and uninfected cells. Binding of the analogue inhibitor 17‐AAG was also demonstrated for the parasite cytoplasmic HSP90 isoform, TA12105. Thus, it was considered likely that both GA and 17‐AGG could have a detrimental effect on the growth and survival of *Theileria* infected leukocytes by targeting and functionally inhibiting parasite and/or host HSP90 isoforms. Therefore, it was of interest to determine whether such inhibition differed between infected or uninfected cell lines.

Cell growth comparisons showed that BL20 cells were sensitive to GA at concentrations that have no or little effect on TBL20 (25 and 50 nM). This differential sensitivity was not apparent at higher concentrations (100 nM) where viability was affected equally in both uninfected and infected cells. One explanation for this is that the increased levels of HSP90 isoforms that are available to bind GA in TBL20 cells effectively titrate out the inhibitory molecule to levels where growth is less adversely affected. Alternatively, the known increase in resistance to cell death of infected cells (Dobbelaere and Kuenzi, 2004) may provide some protection against the outcome of inhibition of HSP90 function at low levels of GA.

For 17‐AAG, the opposite effect was observed, as TBL20 cells were more sensitive to the drug at 2.5 and 5 μM compared to BL20 cells. Recombinant TA12105 clearly had 17‐AAG binding capacity, and it is possible that the other parasite isoforms also bind 17‐AAG. Therefore, it is likely that one or more of these isoforms may be highly sensitive to inhibition by 17‐AAG, and more so than for host isoforms. As parasite HSP90s are likely to be essential for viability, TA10720 for example, being required for optimal functioning of the apicoplast, inhibition will kill the macroschizont and induce death of the host cell after 48 hr, a situation similar to that seen when TBL20 cells are treated with buparvaquone (Guergnon, Dessauge, Langsley, & Garcia, 2003; Kinnaird et al., 2013). Interestingly, the intracellular stage of *Leishmania* was reported to exhibit greater sensitivity to 17‐AAG compared to uninfected host cells (Petersen et al., 2012). Further detailed analysis will be required to identify the *Theileria* isoform with greatest sensitivity to inhibition by 17‐AAG.

The finding that *Theileria*‐infected cells are sensitive to concentrations of 17‐AAG that have limited effect on bovine host cells may provide a starting point for development of novel therapeutics. Such studies are a necessary objective due to emerging field resistance to buparvaquone (Mhadhbi et al., 2015). HSP90 inhibitors have been demonstrated to modulate resistance to antifungal agents such as fluconazole (Cowen & Lindquist, 2005) where high HSP90 is required for azole resistance. This, combined with exploitation of structural differences to specifically target parasite HSP90, may provide a mechanism for dealing with emerging drug resistance, as suggested for *Plasmodium* (Pavithra, Kumar, & Tatu, 2007; Shahinas, Folefoc, & Pillai, 2013). Moreover, based on the degree of similarity across parasite HSP90 isoforms within the ATP binding pocket, the likely 17‐AGG binding site, design, and development of selective inhibitors of *Theileria* HSP90 may provide information of relevance to the other major vector borne Apicomplexa. Such compounds can be tested for pathogen specificity with the convenient comparative cell culture system utilized in this study.

## 4 EXPERIMENTAL PROCEDURES

### 4.1 Parasite stocks and cell culture

All cells were cultured at 37°C in RPMI with 20% foetal calf serum as described previously (Shiels et al., 1992). BL20 is an immortalized bovine lymphosarcoma cell line (Morzaria, Roeder, Roberts, Chasey, & Drew, 1982) routinely used as uninfected bovine cell control. TBL20, parasite‐infected BL20, was previously obtained by *in vitro* infection with T. annulata (strain Hissar) sporozoites (Shiels, McDougall, Tait, & Brown, 1986) providing a system where infected and non‐infected lines represent identical bovine genotypes. D7 is a T. annulata‐infected cloned cell line derived by limiting dilution from a mixed parasite genotype cell line TaA2 that differentiates efficiently to merozoites when cultured in vitro at 41°C (Shiels et al., 1992).

### 4.2 Gene expression and sequence analysis

A published microarray dataset (Pieszko et al., 2015) was used to determine the expression profiles of T. annulata HSP90 isoforms across the parasite life cycle, which included an *in vitro* parasite differentiation time‐course. ArrayStar3 software (DNASTAR) was used to visualize and analyze normalized gene expression values (log_2_). Protein sequences for different parasite HSP90 isoforms were aligned using ClustalW2 (Larkin et al., 2007). The presence or absence of a signal peptide was predicted using SignalP3.0 (Bendtsen, Nielsen, von Heijne, & Brunak, 2004), and apicoplast targeting was predicted using ApicoAP (Cilingir & Broschat, 2015).

### 4.3 Immunofluorescence assay and Western blotting

For immunofluorescence analysis (IFAT), cytospin preparations of cells were fixed in cold 3.7% p‐formaldehyde in phosphate buffered saline (PBS) for 25 min, permeabilized in methanol for 10 min at −20°C then probed with antibody following standard procedures (Schmuckli‐Maurer et al., 2010). Western blotting was carried out using previously described methodology (Kinnaird et al., 2013) with detection by chemiluminescence (SuperSignal West Pico Chemiluminescent Substrate, Pierce). Semi‐quantitation of the X‐ray film signal was carried out using an Alpha Innotec Fluorochem 5500 Imager and Spot Denso tool.

### 4.4 Antibodies

In order to carry out further characterization and to differentiate between host and parasite cytosolic Hsp90, a peptide sequence located near the N‐terminus, KDPKQIEDQPDYYI that was unique to TA12105, was selected. The alignment of the N‐terminal protein sequences of bovine and T. annulata TA12105 protein sequences illustrates high sequence similarity, together with the divergent peptide sequence that was used as the immunogen (Supplementary Figure 1). BLAST of the bovine genome sequence indicated this peptide sequence is not present in the host. Peptide synthesis, immunization, and affinity purification were carried out by GenScript.

For TA06470, generation of protein for antisera production utilized the C‐terminal portion from residue 824 to the end, expressed as a GST fusion protein. The corresponding portion of the encoding gene was cloned into the expression vector pGEX5x‐2 using EcoRI/XhoI sites at the 5´ and at the 3´ ends, respectively. The primer sequences used were as follows: forward, 5´‐ CGGAATTCTATTGTACAATGCAGCCAAGTTA‐3´ and reverse, 5´‐CGCTCGAGCTATAATTCATCATTGGACCAA‐3´. For expression of TA10720, the region of the encoding gene representing the C‐terminus from residue 739 to the end was cloned into pGEX5x‐2 using BamHI/ XhoI cloning sites. Primers were as follows: forward, 5´‐CGGGATCCACGCACAACATCCAATAATAATCA‐3´ and reverse, 5´‐AGGCTCGAGTTATACAAGGTCTAGGGTGTG‐3´. The template for amplification was T. annulata piroplasm genomic DNA prepared from cloned cell line C9 (Pain et al., 2005). Antiserum production was carried out by the PTU/BS Unit at the Scottish National Blood Transfusion Service, Pentlands Science Park Edinburgh.

Antisera were used at the following dilutions for both IFAT and Western blot: TA12105, 1 in 1000 dilution; anti‐serum to TA10720 and TA06470, both at 1500 dilution. Commercial anti‐sera against mammalian GRP94 (Sigma Aldrich, G4545) was used at 1 in 500 dilution for IFAT and 1 in 2000 dilution for Western analysis, and antiserum against the cytoplasmic form of mammalian HSP90 (Enzo LifeSciences SPS771) was used at 1 in 1000 dilution in Western blotting. A monoclonal antibody (5E1) that recognizes a major T. annulata merozoite/piroplasm 30 kDa surface antigen (Glascodine et al., 1990) was used at a 1 in 10 dilution of culture supernatant. A polyclonal antibody that recognizes a macroschizont surface protein, anti‐TaSP (Schnittger et al., 2002), was used at 1 in 1000 dilution for immunofluorescence. Purified recombinant TA12105 was detected by Western blotting using an anti‐6× His antibody (Invitrogen).

### 4.5 Cell fractionation

Cells pellets of 5 × 10^6^ cells were washed in PBS then subjected to a sequential extraction procedure using a Subcellular Proteome Extraction kit (S‐PEK; Calbiochem) to enrich for components from the following cellular compartments: cytoplasm, membranes and organelles, nucleus and finally insoluble cytoskeletal proteins such as intermediate filaments. If necessary, 200 μl aliquots were concentrated for gel analysis using a protein precipitation reagent (Calbiochem).

### 4.6 Production of recombinant TA12105 and estimation of binding affinity and activity

Full‐length TA12105 was PCR amplified using forward primer sequence 5´‐GCCGCGGGATCCATGGCATCAAAGGAAGAGACACC and reverse primer 5´‐ GCGGCGGGTACCTCAGTCAACTTCCTCCATTTTGGAG. These included BamHI and KpnI sites respectively for cloning the 2.7 kb PCR product into the expression vector pRSET A (Invitrogen). This added an additional 35 amino acids consisting of a 6× His tag and an Xpress™ epitope to the N‐terminus of the protein product. The recombinant protein was expressed in E. coli BL21 pLysS cells. Immunoblotting and detection with an antibody specific to the histidine tag confirmed a polypeptide of the predicted size for recombinant TA12105 which was then affinity purified on Ni‐NTA beads (Qiagen). Purity was assessed by 10% SDS‐polyacrylamide gel electrophoresis and Coomassie Blue staining.

The binding affinity of recombinant TA12015 for ATP and 17‐AAG was measured using a fluorescence quenching binding assay as previously described (Pallavi et al., 2010). Briefly, the assay buffer for ATP binding was 40 mM Tris–HCl pH 7.5, 100 mM KCl, 5 mM MgCl_2_, 10 mM DTT with 50 μg purified His tagged TA12105. Intrinsic tryptophan fluorescence was measured by scanning the emission spectrum in the wavelength range of 300–400 nM using an excitation wavelength of 280 nM. The maximum wavelength (λ max) for intrinsic fluorescence was observed to be 340 nM. Change in intrinsic fluorescence in the presence of ligands was plotted against ligand concentration to calculate dissociation constants. Data were analyzed using GraphPad® Prism 5 statistical software.

ATPase activity of recombinant His‐tagged TA12015 was analyzed as carried out previously (Singh, Shah, & Tatu, 2014) by measuring ATP hydrolysis rate with ɤ‐P^32^‐ATP as a tracer. The concentration of cold ATP ranged from 50 to 4000 μM. As a control, 17‐AAG was used at a concentration of 300 μM to correct for any possible non‐specific background ATPase activity. This value was subtracted from the total activity and the remaining activity used to determine ATP hydrolysis rate. Data analysis utilized GraphPad® Prism 5 and Michaelis–Menten kinetics. The ATPase inhibition assay was carried out using the same methodology with a fixed concentration of ATP (2 mM) and a range of different 17‐AAG concentrations (2.5 to 50 μM). Percent residual ATPase activity was plotted against log 17‐AAG concentration to determine the IC_50_.

### 4.7 Geldanamycin affinity

Cell extracts were prepared largely according to (Moulick et al., 2011). Briefly, protein extracts were prepared from 3 to 4 × 10^7^ cells in a lysis buffer containing 20 mM Hepes (pH 7.4), 5 mM MgCl_2_, 50 mM KCl, 0.01% NP‐40, and protein inhibitor cocktail, freeze thawed three times then gently sonicated to shear DNA. Extracts were centrifuged at 14,000 RPM for 10 min and the supernatants used in the GA binding assay. Typically, the protein concentrations of the extracts were 10 mg/ml, and 750 μg were used in a binding assay. Binding of HSP90 from cells extracts was carried out using biotinylated geldanamycin (InvivoGen), pre‐bound to streptavidin magnetic beads (Dynabeads® MyOne™ Streptavidin T1, Invitrogen). Binding of biotinylated GA to Streptavidin magnetic beads was carried out according to the manufacturer's protocol except that biotinylated GA was solubilised in DMSO and incubated with beads that had been washed in lysis buffer, in the ratio 5 nmol GA to 100 μl beads. Dynabeads exposed to DMSO alone were used as a negative control.

### 4.8 HSP90 inhibitor treatment of cell cultures

For each condition, cultures were set up in triplicate at 1.4 × 10 ^5^ cells/ml. Geldanamycin and 17‐AAG (InvivoGen) were solubilised in DMSO and added to cultures at the concentrations detailed in Results with DMSO used as control. Data were expressed as percent survival after drug treatment compared to the respective DMSO control when counted at 48 hr, using Trypan Blue exclusion to assess cell viability and a haemocytometer.

### 4.9 Sequence analysis

Amino acid sequences representing HSP90 isoforms were downloaded from the NCBI database for a number of Apicomplexan species including *Theileria parva*, *Theileria orientalis*, *Babesia bovis*, *Babesia bigemina*, *Babesia equi*, *Plasmodium falciparum*, *Plasmodium vivax*, *Plasmodium chabaudi*, *Toxoplasma gondii*, and Cryptosporidium parvum. The accession numbers for these sequences are detailed in Supplementary Table 1B. A maximum likelihood phylogenetic tree was constructed using RAxML (Stamatakis, 2014), and bootstrapping was performed to assessed tree stability using 100 iterations. The resulting bipartitions tree was visualized using FigTree (http://tree.bio.ed.ac.uk/software/figtree/).

## Supporting information

Supporting info item

Supporting info item

Supporting info item

Supporting info item

Supporting info item

Supporting info item
