# Supplementary material for: Characterization of HSP90 isoforms in transformed bovine leukocytes infected with Theileria annulata
Source: Cell Microbiol. 2016 Oct 20;19(3):e12669. doi: 10.1111/cmi.12669 (PMC5333456; doi:10.1111/cmi.12669)
Supplement: Supplementary file 1 — Supporting info item [file CMI-19-na-s001.pdf]

```
Bos_Hsp90alpha      MPEETQAQDPPMEEEEVETFAFQAEIAQLMSLIINTFYSNKEIFLRELISNSSDALDKIR 60
Bos_Hsp90beta       MPEEVHHG-----EEEVETFAFQAEIAQLMSLIINTFYSNKEIFLRELISNASDALDKIR 55
TaHsp90             MASKEETP-------DQEVYAFNADISQLLSLIINAFYSNKEIFLRELISNASDALEKIR 53
                    *..: .         : *.:**:*:*:**:*****:***************:****:***

Bos_Hsp90alpha      YESLTDPSKLDSGKELHINLIPNKQDRTLTIVDTGIGMTKADLINNLGTIAKSGTKAFME 120
Bos_Hsp90beta       YESLTDPSKLDSGKELKIDIIPNPQERTLTLVDTGIGMTKADLVNNLGTIAKSGTKAFME 115
TaHsp90             YEAIKDPKQIEDQPDYYIRLYADKNNNTLTIEDSGIGMTKADLVNNLGTIAKSGTRAFME 113
                    **::.**.:::.  :  * : .: ::.***: *:*********:***********:****

Bos_Hsp90alpha      ALQAGADISMIGQFGVGFYSAYLVAEKVTVITKHNDDEQYAWESSAGGSFTVR-TDTGEP 179
Bos_Hsp90beta       ALQAGADISMIGQFGVGFYSAYLVAEKVVVITKHNDDEQYAWESSAGGSFTVR-ADHGEP 174
TaHsp90             ALQAGSDMSMIGQFGVGFYSAYLVADKVTVVSKNNADDQYVWESSASGHFTVKRDDSHEP 173
                    *****:*:*****************:**.*::*:* *:**.*****.* ***:  *  **

Bos_Hsp90alpha      MGRGTKVILHLKEDQTEYLEERRIKEIVKKHSQFIGYPITLFVEKERDKEVSDDEAEEKE 239
Bos_Hsp90beta       IGRGTKVILHLKEDQTEYLEERRVKEVVKKHSQFIGYPITLYLEKEREKEISDDEAEEEK 234
TaHsp90             LKRGTRLILHLKEDQTEYLEERRLKELVKKHSEFISFPISLSVEKTQETEVTDDEAEPEE 233
                    : ***::****************:**:*****:**.:**:* :** ::.*::***** ::

Bos_Hsp90alpha      DKEEEKEKEEKESDDKPEIEDVGSDE-EEEEKKDGDKKKKKKIKEKYIDQEELNKTKPIW 298
Bos_Hsp90beta       G---EKEEEDKDDEEKPKIEDVGSDE-EDDSGKD-KKKKTKKIKEKYIDQEELNKTKPIW 289
TaHsp90             EKKLEEEDKDKEEKVEDVTDEKVTDVTEEEEKKEEKKKKKRKVTNVTREWEMLNKQKPIW 293
                        *:*.::*:.. :   ::  :*  *::. *: .***.:*:.:   : * *** ****
```
