# Supplementary material for: Characterization of HSP90 isoforms in transformed bovine leukocytes infected with Theileria annulata
Source: Cell Microbiol. 2016 Oct 20;19(3):e12669. doi: 10.1111/cmi.12669 (PMC5333456; doi:10.1111/cmi.12669)
Supplement: Supplementary file 2 — Supporting info item [file CMI-19-na-s002.pdf]

TA12105 Group 1 orthologues - CLUSTAL 2.1 multiple sequence alignment

```
TpHSP90_1                 MTSKDETPDQEVYAFNADISQLLSLIINAFYSNKEIFLRELISNASDALEKIRYEAIKDPKQIEDQPDYYIRLYADKNNNTLTIEDSGIGMTKADLVNNLGTIAKSGTRAFMEALQAGSD
TaHSP90_1                 MASKEETPDQEVYAFNADISQLLSLIINAFYSNKEIFLRELISNASDALEKIRYEAIKDPKQIEDQPDYYIRLYADKNNNTLTIEDSGIGMTKADLVNNLGTIAKSGTRAFMEALQAGSD
ToHSP_1                   MASKEETKDQEVYAFNADISQLLSLIINAFYSNKEIFLRELISNASDALEKIRYEAIKDPKKIEDQPDYYIRLYADKNANTLTIEDSGIGMTKADLVNNLGTIAKSGTRAFMEALQAGSD
BeqHSP90_1                -MSSDKPADQEVYAFNADISQLLSLIINAFYSNKEIFLRELISNASDALEKIRYEAIKDPKQIEAQPEYYIRLTADKASNTLTIEDSGIGMTKADLINNLGTIAKSGTRAFMEALQAGSD
BBovHSP90_1               ----MATAQQETYAFNADISQLLSLIINAFYSNKEIFLRELISNASDALEKIRYEAIKDPKQVEDFPEYQISLSADKTNKTLTIEDTGIGMTKTDLINNLGTIAKSGTKAFMEAIQAGAD
BbigHSP90_1               ----MATEGQETYAFNADISQLLSLIINAFYSNKEIFLRELISNASDALEKIRYEAIKDPKQVEDFPEYQISLSVDKANKTLTIEDTGIGMTKADLINNLGTIAKSGTKAFMEAIQAGAD
Pf_XP_001348998.1         -------MSTETFAFNADIRQLMSLIINTFYSNKEIFLRELISNASDALDKIRYESITDTQKLSAEPEFFIRIIPDKTNNTLTIEDSGIGMTKNDLINNLGTIARSGTKAFMEAIQASGD
PCHAS_080600              -------MSKETFAFNADIRQLMSLIINTFYSNKEIFLRELISNASDALDKIRYESITDTQKLQAEPEFFIRIIPDKTNNTLTIEDSGIGMTKNDLINNLGTIARSGTKAFMEAIQASGD
PVX_087950                -------MSKETFAFNADIRQLMSLIINTFYSNKEIFLRELISNASDALDKIRYEAITDTQKLSAEPEFFIRIIPDKTNNTLTIEDSGIGMTKNDLINNLGTIARSGTKAFMEAIQASGD
                                    *.:****** **:*****:******************:*****:*.*.:::.  *:: * :  **  :******:****** **:*******:***:*****:**..*

TpHSP90_1                 MSMIGQFGVGFYSAYLVADKVTVVSKNNADDQYVWESTASGHFTVKKDDSHEPLKRGTRLILHLKEDQTEYLEERRLKELVKKHSEFISFPISLSVEKTQETEVTDDEAELDEDKKPEEE
TaHSP90_1                 MSMIGQFGVGFYSAYLVADKVTVVSKNNADDQYVWESSASGHFTVKRDDSHEPLKRGTRLILHLKEDQTEYLEERRLKELVKKHSEFISFPISLSVEKTQETEVTDDEAEPEEEKKLEEE
ToHSP_1                   MSMIGQFGVGFYSAYLVADKVTVVSKNNDDDQYVWESSASGHFTVKKDDSHEPLKRGTRLILHLKEDQTEYLEERRLKDLVKKHSEFISFPISLSVEKTEETEVTN-------------
BeqHSP90_1                MSMIGQFGVGFYSAYLVADKVTVVSKNNDDEQHIWESTASGHFTITKDETGEKLARGTKLILHLKEDQTEYLEERRLKELVKKHSEFISFPISLSVEKTHETEVTDDEAEEEKADDAEKP
BBovHSP90_1               MSMIGQFGVGFYSAYLVADKVTVVSKNNNDDQYVWESNASGHFTVTKDESEDQLKRGTRLILHLKDDQSEYLEERRLKELVKKHSEFISFPIRLSVEKTTETEVTDDEAEPTEAESKPEE
BbigHSP90_1               MSMIGQFGVGFYSAYLVADKVTVVSKNNNDDQYMWESSASGHFTVTKDESGEQLKRGTRLILHLKDDQTEYLEERRLKDLVKKHSEFISFPIRLSVEKTTETEVTDDEAEPAADSETKEE
Pf_XP_001348998.1         ISMIGQFGVGFYSAYLVADHVVVISKNNDDEQYVWESAAGGSFTVTKDETNEKLGRGTKIILHLKEDQLEYLEEKRIKDLVKKHSEFISFPIKLYCERQNEKEITASEEEEGEGEGEREG
PCHAS_080600              ISMIGQFGVGFYSAYLVADHVVVISKNNDDEQYVWESAAGGSFTVTKDETNEKIGRGTKIILHLKEDQLEYLEEKRIKDLVKKHSEFISFPIKLYCERQNEKEITESEDEEAQDGEKKEG
PVX_087950                ISMIGQFGVGFYSAYLVADHVVVVSKNNDDEQYVWESAAGGSFTVTKDETNEKMGRGTKIILHLKDDQLEYLEEKRIKDLVKKHSEFISFPIKLYCERQNEKEITASEDEAEEEDAEGEK
                          :*****************:*.*:**** *:*::*** *.* **:.:*:: : : ***::*****:** *****:*:*:************ *  *:  *.*:*

TpHSP90_1                 ----------------------------------KPKDDKVEDVTDEKVTDVTDEEEKKEEKKKKKRKVTNVTREWEMLNKQKPIWMRLPSEVTNEEYAAFYKNLTNDWEDHLAVKHFSV
TaHSP90_1                 D---------------------------------KDKEEKVEDVTDEKVTDVTEEEEKKEEKKKKKRKVTNVTREWEMLNKQKPIWMRLPTEVTNEEYASFYKNLTNDWEDHLAVKHFSV
ToHSP_1                   -----------------------------------------------------------------------VKREWEMLNKQKPIWMRQPSEVTNEEYASFYKNLTNDWEDHLAVKHFSV
BeqHSP90_1                --------------------------------------KVEEVDDESKDKVEDVTEAEESKEKKKKRKVQSVTREWEMLNKQKPIWMRLPSEVTNEEYASFYKNITNDWEDHLAVKHFSV
BBovHSP90_1               ----------------------------------KITDVTEEEEEKEKE----AEKDGEEKTEKKKRKVTNVTREWEMLNKQKPIWMRLPTEVTNEEYASFYKNLSNDWEDHLAVKHFSV
BbigHSP90_1               ----------------------------------KIKDVTDEVEKEGEEDKDGEDKEGEKSAEKKKRKVTSVTREWEMLNKQKPIWMRLPTEVTHEEYASFYKNLCNDWEDHLAVKHFSV
Pf_XP_001348998.1         EEEEEKKKKTGEDKNADESK--EENEDEEKKEDNEEDDNKTDHPKVEDVTEELENAEKKKK-EKRKKKIHTVEHEWEELNKQKPLWMRKPEEVTNEEYASFYKSLTNDWEDHLAVKHFSV
PCHAS_080600              EDAEKK--------------------------EDGEQKDGEDRPKVEDVTEELENEEKKKK-EKRKKKIHTVEHEWEELNKQKPLWMRKPEEVTNEEYASFYKSLTNDWEDHLAVKHFSV
PVX_087950                KKKEGKDQLDDGDKQAQEGEGADNKEKKEHNEEDEDKEKGEDHPKVEDVTEELENAEKKKKKEKKKKKIHTVEHEWEELNKQKPLWMRKPEEVTNEEYASFYKSLTNDWEDHLAVKHFSV
                                                                                 * :*** ******:*** * ***:****:***.: **************

TpHSP90_1                 EGQLEFKALLFVPRRAPFDMFESRKKKNNIKLYVRRVFIMDDCEELIPEWLSFVKGVVDSEDLPLNISRETLQQNKILKVIRKNLVKKCLELFNELTEKKEDFKKFYEQFSKNLKLGIHE
TaHSP90_1                 EGQLEFKALLFVPRRAPFDMFESRKKKNNIKLYVRRVFIMDDCEELIPEWLSFVKGVVDSEDLPLNISRETLQQNKILKVIRKNLVKKCLELFNELTEKKEDFKKFYEQFSKNLKLGIHE
ToHSP_1                   EGQLEFKALLFVPKRAPFDMFESRKKKNNVKLYVRRVFIMDDCEELIPEWLSFVKGVVDSEDLPLNISRETLQQNKILKVIRKNLVKKCLELFNELTEKKEDFKKFYEQFSKNLKL-IHE
BeqHSP90_1                EGQLEFKALLFIPKRAPFDMFESRKKKNNIKLYVRRVFIMDDCEELIPEWLSFVKGVVDSEDLPLNISRETLQQNKILKVIRKNLVKKCLELFNELTEKKEDFKKFYEQFNKNLKLGIHE
BBovHSP90_1               EGQLEFKAILFVPKRAPFDMFENRKKKNNIKLYVRRVFIMDDCDELIPEWLGFVKGVVDSEDLPLNISREVLQQNKILKVIRKNLVKKCLELFSELTEKKEDFKKFYEQFSKNLKLGIHE
BbigHSP90_1               EGQLEFKALLFIPKRAPFDMFESRKKKNNIKLYVRRVFIMDDCEELIPEWLGFVKGVVDSEDLPLNISREILQQNKILKVIRKNLVKKCLELFAELTEKKEDFKKFYEQFSKNLKLGIHE
Pf_XP_001348998.1         EGQLEFKALLFIPKRAPFDMFENRKKRNNIKLYVRRVFIMDDCEEIIPEWLNFVKGVVDSEDLPLNISRESLQQNKILKVIKKNLIKKCLDMFSELAENKENYKKFYEQFSKNLKLGIHE
PCHAS_080600              EGQLEFKALLFIPKRAPFDMFENRKKRNNIKLYVRRVFIMDDCEEIIPEWLNFVKGVVDSEDLPLNISRESLQQNKILKVIKKNLIKKCLDMFAELAENKDNYKKFYEQFSKNLKLGIHE
PVX_087950                EGQLEFKALLFIPKRAPFDMFENRKKRNNIKLYVRRVFIMDDCEEIIPEWLNFVKGVVDSEDLPLNISRESLQQNKILKVIKKNLIKKCLDMFSELAENKDNYKKFYEQFSKNLKLGIHE
                          *******:**:*:********.***:**:************:*:*****.*****************  *********:***:****::* **:*:*:::*******.***** ***
```

```
TpHSP90_1           DNANRSKIAELLRFETTKSGDELVSLKEYVDRMKSDQKYVYYITGESKQSVASSPFLETLRSRDYEVLYMTDPIDEYAVQQIKEFEGKKLKCCTKEGLDLDEGEDEKKSFEALKEEMEPL
TaHSP90_1           DNANRSKIAELLRFETTKSGDELVSLKEYVDRMKSDQKFVYYITGESKQSVASSPFLETLKARDYEVLYMTDPIDEYAVQQIKEFEGKKLKCCTKEGLELDEGEDEKKSFEALKEEMEPL
ToHSP_1             DNANRTKISELLRFETTKSGDEMVSLKEYVDRMKTDQKFIYYITGESKQAVSTSPFLESLKARDYEVIYMTDPIDEYAVQQIKEFEGKKLKCCTKEGLDLDGDEEEKKSFEALKTEMEPL
BeqHSP90_1          DNANRTKIAELLRFDTTKSGDEAISLKDYVDRMKADQKFIYYITGESKQSVSSSPFLEALRARDIEVIYMTDPIDEYAVQQIKEFEGKKLKCCTKEGLDLEDPEEEKKSFEALKEEMEPL
BBovHSP90_1         DNTNRNKISELLRYETSKSGDEAISLKEYVDRMKPEQKYIYYITGESKQSVANSPFLECLRSRGIEVIYMTDPIDEYAVQQIKEFEGKKLKCCTKENLELEDTEEERKNFETLEKEMEPL
BbigHSP90_1         DNANRTKIAELLRYETSKSGDEAISLKEYVDRMKPDQKYIYYITGESKQSVANSPFLELLRSKGIEVIYMTDPIDEYAVQQIKEFEGKKLKCCTKENLELEDTEEERKSFETLQKEMEPL
Pf_XP_001348998.1   DNANRTKITELLRFQTSKSGDEMIGLKEYVDRMKENQKDIYYITGESINAVSNSPFLEALTKKGFEVIYMVDPIDEYAVQQLKDFDGKKLKCCTKEGLDIDDSEEAKKDFETLKAEYEGL
PCHAS_080600        DNANRAKITELLRFQTSKSGDEMIGLKDYVDRMKDNQKDIYYITGESINAVSNSPFLEALTKRGFEVIYMVDPIDEYAVQQLKDFDGKKLKCCTKEGLDIEDSEEAKKSFETLKAEYEGL
PVX_087950          DNANRAKITELLRFQTSKSGDEMIGLKEYVDRMKENQKDIYYITGESINAVSNSPFLEALTKKGFEVIYMVDPIDEYAVQQLKDFEGKKLKCCTKEGLDIDDSEEAKKTFETMKAEYEGL
                    **:** **:****::*:***** :.**:****** :** :******* ::*:.***** *  :. **:**.**********:*:*:**********.*:::  *: :* **::: * * *

TpHSP90_1           CKHIKEVLHDKVEKVVCGTRFTDSPCALVTSEFGWSANMERIMKAQALRDSSITSYMLSKKIMEINPRHSIMKELKTRAANDKTDKTVKDLVWLLYDTALLTSGFNLDEPTQFGNRIYRM
TaHSP90_1           CKHIKEVLHDKVEKVVCGTRFTDSPCALVTSEFGWSANMERIMKAQALRDSSITSYMLSKKIMEINPRHSIMKELKARAANDKTDKTVKDLVWLLYDTALLTSGFNLDEPTQFGNRIYRM
ToHSP_1             CKHIKEVLHDKVEKVVCGSRFTDSPCALVTSEFGWSANMERIMKAQALRDSSITSYMLSKKIMEINPRHSIMKELKNRAATDKTDKTVKDLVWLLYDTALLTSGFNLDEPTQFGNRIYRM
BeqHSP90_1          CKLIKEILHDKVEKVTCGKRFTESPCALVTSEFGWSANMERIMKAQALRDSSITSYMVSKKIMEINPKHDIMKELLSRSNSDKTDKTVKDLVWLLYDTALLTSGFNLDEPTQFGNRIYRM
BBovHSP90_1         CRLIKEILHDKVEKVVCGKRFTESPCALVTSEFGWSANMERIMKAQALRDSSFGSFMISKKTMELNPHHSIMKELRQRAETDKSDKTLKDLVWLLYDTAMLTSGFNLDDPTQFGGRIYRM
BbigHSP90_1         CRVIKEILHDKVEKVICGKRFTESPCALVTSEFGWSANMERIMKAQALRDNNFGSFMISKKTMELNPHHSIMKELKQRAETDKSDKTLKDLVWLLYDTAMLTSGFNLDDPTQFGGRIYRM
Pf_XP_001348998.1   CKVIKDVLHEKVEKVVVGQRITDSPCVLVTSEFGWSANMERIMKAQALRDNSMTSYMLSKKIMEINARHPIISALKQKADADKSDKTVKDLIWLLFDTSLLTSGFALEEPTTFSKRIHRM
PCHAS_080600        CKVIKDVLHEKVEKVVVGQRITDSPCVLVTSEFGWSANMERIMKAQALRDNSMTSYMLSKKIMEINARHPIITALKQKADADKSDKTVKDLIWLLFDTSLLTSGFALEEPTTFSKRIHRM
PVX_087950          CKVIKDVLHEKVEKVVVGQRITDSPCVLVTSEFGWSANMERIMKAQALRDNSMTSYMLSKKIMEINARHPIITALKQKADADKSDKTVKDLIWLLFDTSLLTSGFALEEPTTFSKRIHRM
                    *: **::**:*****  * *:*:***.***********************..: *:*:*** **:*.:* *:. *  ::  **:***:***:***:**::***** *::** *. **:**

TpHSP90_1           IKLGLSLDDEEHVEEDSSMPPLDEPVVDSKMEEVD
TaHSP90_1           IKLGLSLDDEEHVEDDSSMPPLDEPVVDSKMEEVD
ToHSP_1             IKLGLSLDDE--EQHEEAMPPLDEPVLDSKMEEVD
BeqHSP90_1          IRLGLSLDDDDLADDSAGLPQLEESVVDSKMEEVD
BBovHSP90_1         IKLGLSLDDEP-TGEDVDLPPLDEVVVDPKMEEVD
BbigHSP90_1         IKLGLSLDDDA-PVEDVEIPSLDEVVVDPKMEEVD
Pf_XP_001348998.1   IKLGLSIDEEENNDIDLPPLEETVDATDSKMEEVD
PCHAS_080600        IKLGLSIDEDDHNDIELPPLEETIEGADSKMEEVD
PVX_087950          IKLGLSIDEEENNDIELPPLEETIDATDSKMEEVD
                    *:****:*::     .           *.******
```
