# Supplementary material for: Characterization of HSP90 isoforms in transformed bovine leukocytes infected with Theileria annulata
Source: Cell Microbiol. 2016 Oct 20;19(3):e12669. doi: 10.1111/cmi.12669 (PMC5333456; doi:10.1111/cmi.12669)
Supplement: Supplementary file 3 — Supporting info item [file CMI-19-na-s003.pdf]

TA10720 Group 2 orthologues - CLUSTAL 2.1 multiple sequence alignment

```
TpHSP90_2          --MNPQLGFRSNYRTNLVNLSHSAFLCLIFFSFITFCYSWTGRLTHTSQLNPYTNYKNNPPP----SSIKNHHEFQTNAFEDLTNKVSGLKNVLQQKFGLSNELSTMDNPQVSAYEGDPS
TaHSP90_2          --MNPRLGFRINYRTNIVNLSHSAFFCLIFFLYITFCYTWTGRVTLKSHLNPYTNYKNNLVP----SSIKNHHEFKSNALEDLTNKVSGLKDVLQQKFGLSNELTTTENAQVSKYEGEPA
ToHSP90_2          --MNPQLGHRHNPYLNLLNRRLTTFLCLIIVVHTRLSCSWTGSPSNISQFHLSNVKTVNSQN----LTLKNYRDLRRNSHNGLEDKVEDLKNVFQQKFGLENELSKMEDPQVSTYEGDET
BeqHSP90_2         MESLALFVPSTNKRTTSTIIRLVIYSYLFVLLKCKPCSPWRGHIPLSSQFPRTGKAFIHPSAGESHNIPKNRRNFKFNALEDANDNVSDLKNVLQDRVGLSSEASKLDSAKVSKFEDSEL
BbovHSP90-2        --MRARILLSSPKGVRWALRVLPLLLCISSTIAFNIKSKDVSSLHSRLQQPQLKLGSGHQSR-----------PLYGFFSSDSSETTDALKGALQPKIGLSAEVNSMDGAKVSRYDDELN
BbigHSP90_2        --MTRAAARAGCRWARCAFVGLLALSLLLVTSAFRPKPKFVSSLQHRRQEPHLQVEGSRRAP-----------PLYGSSADEPSGVTSGLQDALQHKIGLSSELSGMEDAKVTRLDDELG
PVX_118295         ------------MQNARVANKIKLILCLLFAALLKPND-VTEAYNTARNAEKLNYILNYKNA-------------SRYHIDNRINKTFLKKKK----LKGNTLNSFNDDVKTIRED----
PCHAS_131100       ------------MQNAYVSHKTKLMLLFFIVVFLKCNDTIIEAFNFSRSAEKLNYVLNYKNS-------------NKYRIDQNINKSFLKKRQ----FKRNSILGLDNDVQISDGG----
PfHSP90_2          ------------MQNVYVGNKIKFIILYFFCVLFLKDYERSEAFNLARTTEKLNYILNYKTP-------------NRYDLNNNVNKLFFEKQKKKIEFSRKPLNSFNEDVKTIRED----
                                                                                   .         :      .  .      :. :

TpHSP90_2          TPKAPQE-PPEVSLSGEQTYPFQAEVSRVMDIIVNSLYTDRDIFLRELVSNSADALDKRRLKADPE-----------------------------------------EKIPKEAFGGIRIMPNK
TaHSP90_2          EPKKVEE-SQELTLSGEQTYPFQAEVSRVMDIIVNSLYTDRDIFLRELVSNSADALDKRRLKADPE-----------------------------------------EKIPKEAFGGIRIMPNK
ToHSP90_2          LTKSASQ-PEKVEVSGEETYPFQAEVSRVMDIIVNSLYTDKDIFLRELVSNAADALDKRRLKADPE-----------------------------------------EKVPKEAFGGIRIIPDK
BeqHSP90_2         KYETVKVESPKISVSGEVSYPFQAEVSRVMDIIVNSLYTDRDIFLRELVSNSADALDKRRIKADPD-----------------------------------------EKIAKEAFGGIRIIPNK
BbovHSP90-2        ----LDSPSPCVKVENEQTYPFQAEVSRVMDIIVNSLYTDKDIFLRELVSNAADALDKRRIQADPD-----------------------------------------EKVPKESFGGIRIIPDK
BbigHSP90_2        ----LGALGPDVKVQDEQSFPFQAEVSRVMDIIVNSLYTDKDIFLRELVSNAADALDKRRIQADPE-----------------------------------------EKVPKEAFGGIRIFPDK
PVX_118295         ---------MSADSSPVEKYNFKAEVNKVMDIIVNSLYTDKDVFLRELISNASDACDKKRIILQNEKQMKEAQDIANSSVAKSDVEKSTPEGANNNGEVENKEQ-VDEIKKLIIKIKPDK
PCHAS_131100       ---------AQSDDTPVEKYNFKAEVNKVMDIIVNSLYTDKDVFLRELISNASDACDKKRIGLENEKRAEEAQNIVNG-------STSSELASEQKTTEEGNTP-ADNIKKLIIKIKPDK
PfHSP90_2          ---------ISSDSSPVEKYNFKAEVNKVMDIIVNSLYTDKDVFLRELISNASDACDKKRIILENNKLIKDAEVVTNEEIKNETEKEKTENVNESTDKKENVEEEKNDIKKLIIKIKPDK
                                     .: *:***.:************:*:*****:**::** **:*:  : :                                         .     *:* *:*

TpHSP90_2          DLSTLTIEDDGIGMTAEELKTNLGTIAESGTAKFLQQIDTTG--------ENNLIGQFGVGFYSSYLVSNKVEVFSRAYGQEAGPVYRWKSDSNGTYTIGRVENQELNDKFMKSG-TRIV
TaHSP90_2          ELSTLTIEDDGIGMTAEELKTNLGTIAESGTAKFLEQIEITG--------NNNLIGQFGVGFYSSYLVSNKVEVFSRAYGQENGPVYRWKSDSNGTYTIGKVENQELNEKFMKCG-TRIV
ToHSP90_2          NVSTLTIEDDGIGMSAEELKTNLGTIAESGTAKFLKQVSETENDCLDGVEESNLIGQFGVGFYSSYLVSNKVEVYSRAYGKEEGPVYRWKSDSNGTYTIGKVNDQELNEKFMKCG-TRIV
BeqHSP90_2         VANTLTIEDDGIGMTVEELKKNLGTIAESGTAKFLQQYEAGKS-------SSNLIGQFGVGFYSAFLVANTVEVYSRAYGLETGPVHRWKSDTTGTFSVAQVADDSINKSFMPCG-TRIV
BbovHSP90-2        EHNTLTIEDDGIGMTKDELVHNLGTIAESGTAKFLKQLES--GTDTN-----NLIGQFGVGFYSAFLVSNKVEVYSRAYGHEDGGIFRWKSETNGTFSVAQVNDDELQKGFMKCG-TRIV
BbigHSP90_2        EHNTLTIEDDGIGMTKDELVKNLGTIAESGTAKFLKQLETSKSTDTN-----NLIGQFGVGFYSAFLVANRVEVFSRAYGHEDGPIYRWRSDTSGSFSVGQVDDEQVSSSFMKTG-TRIV
PVX_118295         ETKTLTITDNGIGMDKNELINNLGTIAQSGTAKFLKQIEEGKADS-------NLIGQFGVGFYSSFLVSKKVEVFTKKENT----IFRWFSDLNGSFMVNEIKKYEQEYEDIQSSGTKIV
PCHAS_131100       EKKTLTITDNGIGMDKNELINNLGTIAQSGTAKFLKQIEEGKADS-------NLIGQFGVGFYSSFLVSNKVEVFTKKEDR----IFRWFSDLNGSFNVNEIKKYEQEYEDIKTSGTKIV
PfHSP90_2          EKKTLTITDNGIGMDKSELINNLGTIAQSGTAKFLKQIEEGKADS-------NLIGQFGVGFYSSFLVSNRVEVYTKKEDQ----IYRWSSDLKGSFSVNEIKKYDQEYDDIKGSGTKII
                     .**** *:****  .**  ******:*******:* .             ************::**:: ***:::  .     :.** *: .*:: : .: . . .   :  . *:*:

TpHSP90_2          LHLKPECDDYLEDYKLKELLRKYSEFIRFPIQVWVERIEYERVPDD-ATIVDGKPGRYKTVTKKRNEWEVVNTQLPIWRRDQSTIKPEDYISFYKSTFKAYEDPLSYIHFKVEGQVEFTC
TaHSP90_2          LHLKPECDDYLEDYKLKELLRKYSEFIRFPIQVWVERIEYERVPDD-ATIVDGKAGRYKTVTKKRHEWEVVNTQLPIWRRDQATIKPEDYISFYKSTFKAYEDPLSYIHFKVEGQVEFTC
ToHSP90_2          LHLKPECDDYLEDYKLKELLRKYSEFIRFPIQVWVERIEYERVPDD-TTMVDGKPGRYKTVTKKRHEWEVVNTQLPIWRRDQSSIKPEDYVSFYKSTFKAYEDPLSYIHFRVEGQVEFTC
BeqHSP90_2         LHLKPECDDYLEDYKLKELLRKYSEFVRFPIQVWVEKVEYERVPDE-TTAVEGKPGRYKTVTKKRNEWEVVNTQMPIWRRSQDDIRPEDYVSFYKSTFKAYDDPLSYIHFKVEGQVEFSC
BbovHSP90-2        LHIKPECDDYLEDYKIKELLRKYSEFVRFPIQVWVEKVEYERVPDE-STAVEGKPGRYKTISKKRHEWEHVNTQIPIWRRDQADVKPEDYVSFYKSTFKAYDDPLSYIHFKVEGQVEFSC
BbigHSP90_2        LHIKPECDDYLEDYKIKELLRKYSEFVRFPIQVWVEKVEYERVPDE-STAVEGKPGRYKTISKKRHEWEHVNTQIPIWRRDQADIKPEDYVSFYKSTFKAYDDPMSYIHFRVEGQVEFSC
PVX_118295         LHLKEECDEYLEDYKLKELIKKYSEFIKFPIEIWSEKIDYERVPDDSVSLKDGDKMKMKTITKRYHEWEKINVQLPIWKQDEKKLTENDYYSFYKNTFKAYDDPLAYVHFNVEGQISFNS
PCHAS_131100       LHLKEECDEYLEDYKLKELIKKYSEFIKFPIEIWSEKIDYERVPDDSVSLKDGDKMKMKTITKRYHEWEKINVQLPIWKQDEKQLTENDYYSFYKNTFKAYDDPLAYVHFNVEGQISFNS
PfHSP90_2          LHLKEECDEYLEDYKLKELIKKYSEFIKFPIEIWSEKIDYERVPDDSVSLKDGDKMKMKTITKRYHEWEKINVQLPIWKQDEKSLTENDYYSFYKNTFKAYDDPLAYVHFNVEGQISFNS
                   **:* ***:******:***::*****::***::* *:::******:  :  :*.  : **::*: :*** :*.*:***::.:  :  :** ****.*****:**::*:**.****:.*..
```

```
TpHSP90_2          LLFVPGTLPWELSRNMFDDESRGIRLYVKRVFINDKFSESIPRWLTFVRGVVDSDELSLNVGREYLQRSKALTVINKRIASKAIDMLKNLRN-NKVRFEKFSENFGKYIKIGVVEDRDNQ
TaHSP90_2          LLFVPGTLPWELSRNMFDEESRGIRLYVKRVFINDKFSESIPRWLTFVRGVVDSDELSLNVGREYLQRTKALTVINKRIASKAIDMLKNLKN-NKARFQKFCENYGKYIKIGVVEDRDNQ
ToHSP90_2          LLFVPGSVPWELSRNMFDEESRGIRLYVKRVFINDKFSESIPRWLTFVRGVVDSDELSLNVGREYLQRSKALTVINKRIAAKAIDMLKNLKNSNKARFERFTENFGKYIKIGVVEDRDNQ
BeqHSP90_2         LLFVPGSLPWELSRNMFDEQSRGIRLYVKRVFINDKFTEAIPRWLTFIRGVVDSDELSLNVGREYLQKSKALTVINKRISTKAVDMLKNLKASSPERFKKFTDNFGKYIKIGIVEDRDNQ
BbovHSP90-2        LLFVPGSLPWELSRNMFDDQSRGIRLYVKRVFINDKFSEAVPRWLTFVRGVVDSDELALNVGREYLQRSKALTIINKRIAIKAIDMFKNLQAANPDRFKKFTDNFGKYIKIGVVEDRENQ
BbigHSP90_2        LLFVPGSLPWELSRNMFDDESRGIRLYVKRVFINDKFSEAVPRWLTFVRGVVDSDELALNVGREYLQRSKALTVINKRIATKALDMLKNLKAANPERFRKFSDHFGKYIKIGVVEDRENQ
PVX_118295         ILYIPGSLPWELSKNMFDEESRGIRLYVKRVFINDKFSESIPRWLTFLRGIVDSENLPLNVGREILQKSKMLSIINKRIVLKSISMMKGLKETGGEKWNKFLNTFGKYLKIGVVEDKENQ
PCHAS_131100       ILYIPGSLPWELSKNMFDEESRGIRLYVKRVFINDKFSESIPRWLTFLRGIVDSENLPLNVGREILQKSKMLSIINKRIVLKTINMMRGLKETGGEKWNKFLNTFGKYLKIGVVEDKENQ
PfHSP90_2          ILYIPGSLPWELSKNMFDEESRGIRLYVKRVFINDKFSESIPRWLTFLRGIVDSENLPLNVGREILQKSKMLSIINKRIVLKSISMMKGLKETGGDKWTKFLNTFGKYLKIGVVEDKENQ
                   :*::**::*****:****::*****************:*::******:**:***::*.****** **::* *::*****  *::.*::.*:  .  :: :* : :***:***:***::**

TpHSP90_2          QELASLTTFKSTK--EKSTTLDDYIQRMKKDQPAIYYISADSEQSAQNSPSLEKFNQLDYEVLYSLEPVDEFCLSSLMATKYKGIMIQDVNKHDVKIGENQTLSETSKTEGTTTETPQG-
TaHSP90_2          QELASLTTFRSTK--EKSTTLDDYIQRMKKEQPAIYFISADSEQSAQNSPSLEKFNQLDYEVLYSLEPVDEFCLSSLMASKYKGIMIQDVNKHDVKIGESKSLTETSTTEGTKTEAPRG-
ToHSP90_2          QELASLVMFRSSK--EKKTTLDEYVQRMKKDQPAIYFIAADSEQSAENSPSLEKFNQLDYEVLYSLEPIDEFCLSSLMASKYKGIMIQDVNKHDVKLGESAESGSGSGSADGKGSDGIGG
BeqHSP90_2         QELASLTSFYSSHGGNDRVSLDEYIERMKPNQPAIYYVTADSIQSAKNLPSLEKLKALDYEVLFSLEPLDEFCLASIAASKYKNIMVVDVNKSDLKLQEPTEEEQSKEGEKKNP------
BbovHSP90-2        QDLASLVSFWSTKSGQDRVTLDEYIKRMKPNQPAIYYLTADNLQAAQSSPSLEKLKALDYEVFYALEPIDEFCFSSLTASKYKNIMVLDVNKSDLKLSEADNKQESAQKD----------
BbigHSP90_2        QELASLVTFWSTKSGQNRVSLDEYVGRMKPEQSAIYYLTADDIKSAQSSPSLEKLKALDYEVLYALEPIDEFCLSSLTAAKYKNITVLDVNKSDLKLKEAADG-EPSQKQ----------
PVX_118295         EEIASLVEFYSINSGDKKIDLDTYIENMKPDQKCIYYISGENKKTAQNSPSLEKLKALNYDVLFSLEPIDEFCLSSLTVNKYKGYDVLDVNKADLKLKKENDQNQTDSLDKLK-------
PCHAS_131100       EEIASLVEFYSINSGDKKIDLDTYIENMKSDQKCIYYISGENKKTAQNSPSLEKLKALNYDVLFSLEPIDEFCLSSLSVNKYKGYDVLDVNKADLKLKTENDQNKSDNINKLK-------
PfHSP90_2          EEIASLVEFYSINSGDKKTDLDSYIENMKEDQKCIYYISGENKKTAQNSPSLEKLKALNYDVLFSLEPIDEFCLSSLTVNKYKGYEVLDVNKADLKLKKENDQNKSDSLDKQK-------
                   :::***. * * :  :.   ** *: .** :* .**:::.:. ::*:. *****:: *:*:*:::***:****::*: . ***.  : **** *:*:       .

TpHSP90_2          -----------------DFEMLCNWIKTTFPDRVQEVKVSNRLVESPALLVQTDFGLSPSMQKYMRQQATSVGMNETEMFGTSLVSKPVLEINAQHPIIVNLEKMLKVDALRDRMKEIAA
TaHSP90_2          -----------------DFEMLCNWLKTTFPERVQEVKVSSRLVESPALLVQADFGLSPSMQKYMRQQATSAGMNETEMFGTSLVSKPVLEINAQHPIIINLEKMLKVDSLRDRMKEIAA
ToHSP90_2          GASGGGASSGGRGGSEADHEMLCTWMKSNFSDRVQEVKISRRLVESPALLVQADFGLSPSMQKYMRQQATSAGMNETELFGTSMVSKPVLELNPDHPIIASLERMLKVDALRDRMKEIAG
BeqHSP90_2         ----------------LEFETLCGWLKGLFHDRLHDVKVSTRLVESPALLVQTDFGLSPSMQKYMKQQATSAGMDETELFGTSMVSKPVLEINIDHPIIQHLNEMVKKDKLGDAPRDMAK
BbovHSP90-2        --------------NSVEYETLCGWLKQLFPDDLHDVKVSKRLVESPAILVQTDFGLSPSMQRYMKQQATSAGMNDTELFGTSMVSKPVLEINIDHPIIQHLNMMVKTDKLSDVPRQVAK
BbigHSP90_2        --------------NPVEFETLCGWMKALFPDDLHDVKVSTRLVESPAILVQTDFGLSPSMQRYMKQQATSAGMNDTELFGTSMISKPVLEINIDHPIIQHLNAMVKTDKLSDLPRQVAK
PVX_118295         ----------------MKYEILCRWLHNKFSHKVHEVRISDRLINSPALLVQGEMGISPSMQKYMKQQATAQGMSENEMFGGQSANQPVLEINPNHYIIKQLNHLIQIDKMNSQNSEIAE
PCHAS_131100       ----------------IKYEVLCRWLHNKFSHKIHEVRISDRLIDSPSLLVQGEMGISPSMQKYMKQQATAQGMSENEMFGGQSMNQPVLEINPNHYIIKQLNHLIQMDKMNPKNDEIAE
PfHSP90_2          ----------------MEYEILCRWLHNKFSHKVHEVRISDRLINSPALLVQGEMGMSPSMQKYMKQQATAQGISENEMFGGQSANQPVLEINPNHFIIKQLNHLIQIDKMNLQNSEIAE
                                    ..* ** *::  * . :::*::* **::**::*** ::*:*****:**:****: *:.:.*:** .  .:****:* :* **  *: ::: * :     ::*

TpHSP90_2          QLLDVVSIQGGYSIEDPSSFAKGIIKLMQNEAKHYLTNPSEKTPETNETLSEFAASKPSESCLLNSNTGSSVTDSPKEPVSKVTDTTMSSNKENEKVEQITKEEAQEVSNTLKDNEMGLK
TaHSP90_2          QLLDVVSIQGGYSIEDPSSFAKGIIKLMQNEAKHYLDDPAETTPVTKETLSEFSSSKQNEACELKSNSESNEVDSGSDQVSKVMDKTTSTNLGNEKAEQTTKEQVEELSNTLKDNEMGLK
ToHSP90_2          RLIDVVSIQGGYVVEHPSTFAKGIIKLMQNEASRYLSESEREAEEGGENTEQIAASKSRDPEKETHRPLASEENMNPEDVSTSSSSSD------------------------------LA
BeqHSP90_2         QLLDVVSIQGGYTIEDPSLFAKNVLNLMQREASKYFSEEAAKS-----------------------------------------------------------------------------
BbovHSP90-2        QLLDVVSIQGGYNVKDPTQFARSVLDLMQREAKQFIEKQESA------------------------------------------------------------------------------
BbigHSP90_2        QLLDVVSIQGGYSVKDPTVFARNVLDMMQREAKQFLDAKSASES----------------------------------------------------------------------------
PVX_118295         QIFDIASMQGGYTIDDTGLFAKRVIGMMEKNAQTYLMNVQGNLDSTPSESSPSSSSEPAQNSAQGDSTQGNAGGSNPALDNPSSSSESAGLSEPSADASSTGGFGEERLSASNLDGLGGD
PCHAS_131100       QIFDVASMQGGYTIDDTGRFAKRVIGMMERNAQEYLKDVQDDIDITPSNNSEDTALDSTTPNDESQINPEQSDINQNDSTGDNAS-----------------------------------
PfHSP90_2          QIFDVASMQGGYTIDDTGLFAKRVIGMMEKNAEQYLMNVQSNISNNTLNNNTSGSEMPQNNSPNELQSEMKSTNGIDDNSNISENKINESSSNQNNIGENS-------------------
                   :::*:.*:**** :...  **: :: :*:.:*. ::
```

```
TpHSP90_2         VAKEEFEGGSELVRSPSGPVETHTLNLV----------------------------------
TaHSP90_2         DPKEEFNEGSNLVRSTSGHVGTHTLDLV----------------------------------
ToHSP90_2         SGKEEGTGSPELVGTTSETINDVVQ-------------------------------------
BeqHSP90_2        --------------------------------------------------------------
BbovHSP90-2       --------------------------------------------------------------
BbigHSP90_2       --------------------------------------------------------------
PVX_118295        NASANANANESSNASTNSIGANGLSDSALNGSNMNGLDPNLYNIDRNIFNDKMFSGSDKTVL
PCHAS_131100      --------------------------------------------------------------
PfHSP90_2         ----IAEENNIKNIAESDVNKINLGENDVSQNTMHKQDSGLFNLDPSILNSNMLSGSDKTLL
```
