# Supplementary material for: Characterization of HSP90 isoforms in transformed bovine leukocytes infected with Theileria annulata
Source: Cell Microbiol. 2016 Oct 20;19(3):e12669. doi: 10.1111/cmi.12669 (PMC5333456; doi:10.1111/cmi.12669)
Supplement: Supplementary file 4 — Supporting info item [file CMI-19-na-s004.pdf]

TA06470 Group 3 orthologues - CLUSTAL 2.1 multiple sequence alignment

```
TaHSP90_3         -------MKVSLLLLIKLFFILQLNKGYYFVSCNESDELE----VVED---TVN-------------------------------------------------------------VDEDV
TpHSP90_3         --------MNISLFLLRLIFILYLSRGDNFAFCDESDDLE----VVED---PVS-------------------------------------------------------------FDEIK
ToHSP90_3         -----------MKISIFLRVYLFVYLSSAYISCKKYVDLE----LPGD---LVDPEVNDLEVEHRSSKLSDELSPEDKSDFEDTVDFTETVDLDDSFDLAESPEETPDDSVVVDDEVKVE
BeqHSP90_3        -----MKVTKLALLHIFFVFVLYSPSFVRYTLCEEDVDVE----VTETPEE---------------------------------------------------------------------
BBovHSP90_3       -------MKLSTVYILQCILAISAINYIQYPVCAEDVEDS----IEDIVDEKSE---------------------------------------------------------QPPISIDLR
BBigHSP90_3       -------MRRSFAVMLQCIATIFAVIHFQNVEVFANEEKG----VESSIDSADFGS----------------------------------------------------DNIQPRIHVEST
PVX_123745        -----MKLNRVIPCALLIGALLPSWVPQTFNVLCASDEGK--------------------------------------------------------------------------------
PCHAS_143930      MSLGKMKIKTKYTYAFFVFLIVFNLLSKNNNVFCEDDPSN--------------------------------------------------------------------------------
PfHSP90_3         -----MKLNNIYSFFFLFFVLCVIQENVRRVLCDSSVEGDKGPS----------------------------------------------------------------------------
                                 :

TaHSP90_3         TEEQTPSELSEEELLDRSEDSSVLTSEKLFKDSTKSEKYEYQAEVTRLLDIIVNSLYSSKDIFLRELVSNSADALEKYKITALQKNYRDKDDVELFVRIRSYPKKRLLTIWDNGVGMTKT
TpHSP90_3         VEDEAPAALSEEELLDMSEDSSVLTSEKLFKDSAKSEKYEYQAEVTRLLDIIVNSLYSSKDIFLRELVSNSADALEKYKITALQKNYKDKD-VELFVRIRSYPKKRLLTIWDNGVGMTKS
ToHSP90_3         EEEEPDRELTEEELLDNSDDSSVLTSDKLFRDSTKSEKFEYQAEVTRLLDIIVNSIYSSKDIFLRELVSNSADALEKYKITALQKNYSDKDTVDLFIRVRSYPKKRLLTIWDSGVGMTKN
BeqHSP90_3        --KEEEVKLSEEELLNQAEDPVLLSEDDISKLSKTGEHHEYQAEITRLLDIIVNSLYSSKEIFLRELISNSADALEKYKIFALQNDYEDKG-EELNIKIRAIPNKRILTIMDNGIGMTKH
BBovHSP90_3       DGKAEEETEKPEVEVTEPEDFVSLSDDEMTQAAKHGESHTYQADFARVMDIIVNSLYSNKDVFLRELISNSADALEKYKIVELRENRSESV-DELAIKIRVSKNKRTLTILDTGVGMTKH
BBigHSP90_3       EEDTSDGAADSEVVITEPEDTVSLSDDEMSSAAKHGESHTYQADFARVMDIIVNSLYSKKEVFLRELISNSADALEKYKILELRENRADSG-DELAIRIRASAAKRTLTILDTGVGMTKH
PVX_123745        ------GEEKEKKEETKKDTDNIPEIADNEKPTSGIEQHQYQTEVTRLMDIIVNSLYTQKEVFLRELISNAADALEKIRFMSLSDEKVLGEEKKLEIRISANKEKNILSITDTGIGMTKE
PCHAS_143930      ------ASGGGPKPYVKRDVDMISEIDENEKPTSGIENHQYQSEVTRLLDIIINSLYTQKDVFMRELISNAADALEKIRFLSLSDESVLNDEKKLEIRISANKDKNILSITDTGVGMTKD
PfHSP90_3         ---DDVSDSSGEKKEVKRDRDTLEEIEEGEKPTESMESHQYQTEVTRLMDIIVNSLYTQKEVFLRELISNAADALEKIRFLSLSDESVLGEEKKLEIRISANKEKNILSITDTGIGMTKV
                                    :        .    :   * . **::.:*::***:**:*:.*::*:***:**:****** ::  * .:       .* :::     *. *:* *.*:****

TaHSP90_3         ELMNNLGTIAKSGTANFLDSLSKAGSDPNLIGQFGVGFYSAFLVADTVLVQSKNYDDKQYVWRSSAANNYELYEDTD-NSLGDHGTLITLELREDSTEYLKTDVLENLVKKYSQFVRYPI
TpHSP90_3         ELMNNLGTIAKSGTANFLDSLSKVGNDPNLIGQFGVGFYSAFLVADTVLVQSKNYEDKQYVWRSSAANSYELYEDTD-NSLGDHGTLITLELREDATDYLKTDVLENLVKKYSQFVKYPI
ToHSP90_3         DLMNNLGTIAKSGTANFLDSLSKVGNDPNLIGQFGVGFYSAFLVADTVIVQSKHRDDKQYVWKSSAANSYELFEDSE-NTLGDHGTLITLELREDATEYLKTDVLENLVKKYSQFVKYPI
BeqHSP90_3        DLINNLGTIAKSGTANFLDALNKGEGDASLIGQFGVGFYSAFLVADTVIVQSKHSTDKQYVWKSSADANYELYEDPKGDTLGAHGTLITLKLREDATNYLKPDVLQDLVKKYSQFVKHPI
BBovHSP90_3       ELINNLGTIAKSGTANFIDAITKGENDSNLIGQFGVGFYSVFLVADSVVVQSKHLEDKQYVWKSSADTKYELYEDPKGNTLGEHGTQITLFLREDATEYLEIDKIEELIKKHSQFVRFPI
BBigHSP90_3       ELINNLGTIAKSGTANFVDAISKGENDANLIGQFGVGFYSVFLVADSVVVQSKHWNDKQYVWKSSADTKYELYEDPKGNTLGEHGTQITLFLKEDATEYLEAAKIEELIKKHSQFVRFPI
PVX_123745        DLINNLGTIAKSGTSNFLEAISKSGGDMSLIGQFGVGFYSAFLVADKVIVYTKNNNDEQYIWESTADAKFSIYKDPRGSTLKRG-TRISLHLKDDATNLMNDKKLVDLISKYSQFIQYPI
PCHAS_143930      DLINNLGTIAKSGTSNFLETISKSGGDMSLIGQFGVGFYSAFLVADKVVVYTKNNNDEQYIWESTADAKFSIYKDPRGATLKRG-TRISLHLKEDATNLLNDKKLTDLISKYSQFIQFPI
PfHSP90_3         DLINNLGTIAKSGTSNFLEAISKSGGDMSLIGQFGVGFYSAFLVADKVIVYTKNNDDEQYIWESTADAKFTIYKDPRGATLKRG-TRISLHLKEDATNLLNDKKLMDLISKYSQFIQFPI
                  :*:***********:**::::.*  .* .***********.*****.*:* :*:  *:**:*.*:*  .: :::*.   :*    * *:* *::*:*: ::   : :*:.*:***::.**

TaHSP90_3         QLYKK-------------------------------------LKDKQEVGWVKVNETQQIWTRNKNTITEEEYNQFYKTISGKNDEPLTHVHFTAEGDVDFKALLYIPSSPPGMYFSTES
TpHSP90_3         QLYKK-------------------------------------LKDKQELGWVKVNETQQIWTRNKNTITEQEYNEFYKTISGKTDEPLAHVHFTAEGDVDFKALLYIPSSPPAMYFSSES
ToHSP90_3         QLYKK-------------------------------------LGEKQELGWVSVNETQQIWTRSKNTITEKEYNDFFKKISGKDEEPLAHVHFTAEGDVDFKALLYIPSSPSAMYFTTDT
BeqHSP90_3        KLYKL-------------------------------------SKDGDSVDWAVVNDVPPIWTRDKTTITPEEYISFYKAISGHTEDPLTHIHFSAEGDVDFKALLYIPARPANIYFDSNS
BBovHSP90_3       YVLKA-------------------------------------VKGEPEAKWQHVNDIKPIWARDKSEITEDEYTAFYKAISGSTSKPLAHIHFVAEGDIDFRALLYIPERPKSAYFDNED
BBigHSP90_3       YVLTV-------------------------------------DKESKEAKWKHVNDVKPIWARDKSEITDEEYTEFYQAISGARSKPLAHIHFVAEGDVEFRSLLFIPERPKSSYFDTDD
PVX_123745        YLLHENVYTEEVLADIAKEMENDPNYDSVKVEESDDPNKKTRTVEKKVKKWKLMNEQKPIWLRPPKELTDEDYKKFFSVLSGFNDEPLYHIHFFAEGEIEFKCLIYIPSRAPSINDHLFT
PCHAS_143930      YLLYENVYTEEVLADIAKEMENDPNYDSVKVEEPDDPNKKTRTVEKRVKQWKLMNEQKPIWLRPPKELTEEDYKNFYSVLTGYNDEPLYQIHFFAEGEIEFKCLIYIPSKAPSINDQMFS
PfHSP90_3         YLLHENVYTEEVLADIAKDMVNDPNYDSVKVEETDDPNKKTRTVEKKVKKWTLMNEQRPIWLRSPKELKDEDYKQFFSVLSGYNDQPLYHIHFFAEGEIEFKCLIYIPSKAPSMNDQLYS
                   :                                                *  :*:   ** *  . :. .:*  *:. ::*  ..** ::** ***:::*:.*::**  .
```

```
TaHSP90_3         VGHNVKLYSRRVLVSQEMKDFIPRYLFSIYGVVDSDSFPLNVSREYLQQSKLVKLIGKKVVRTVLDTLYDVMKKSQEDVKETEAELEKVKAVKEEKEWNSYKKDWKKRNEVEYKEFEESA
TpHSP90_3         VGHNVKLYSRRVLVSQEMRDFIPRYLFSVYGVVDSDSFPLNVSREYLQQSKLVKLIGKKVVRTVLDTLYDVMKKSYEDVKEVEDELEKVKSVKEEKEWNTYKKDWKKRNDKEFKEFELSS
ToHSP90_3         VNHNVKLYSRRVLVSENLRDFIPRYLFSVYGVVDSDSFPLNVSREYLQQSKLVKVIGKKVVRTVLDTLFDVMKKSEDDVKEVSEELEKVKAVNGESEWNSYKKEWKKRDESGYKSFVEEL
BeqHSP90_3        KEHNVKIYSRRVLVSEELPDFIPRYLFSIYGVVDSDSFPLNVSREHLQQSKLIKVIGKKIVRTVLDTLLDLMKKSDKSKKALKEELEAETDEE---------------------------
BBovHSP90_3       VGHHVKIYARRVLVSDSLPNFLPRYLYSLHGVVDSDNFPLNVSREHLQQSKMIKIIAKKIVRSVLTTLENLMKESMENKKQLREELESETDEE---------------------------
BBigHSP90_3       VGHQVKIYARRVLVSDRLPDFLPRYLYSICGVVDSDNFPLNVSREHLQHSKMIKIIGKKIVRSVLSTLQNLMKESFDSKKKLQEEIDAETDEE---------------------------
PVX_123745        KQNSIKLYVRRVLVADEFVEFLPRYMSFIKGVVDSDDLPLNVSREQLQQNKILKAVSKRIVRKILDTFRTLYTSGKKNKEDLRAQLAKETDEE---------------------------
PCHAS_143930      KQSSIKLYVRRVLVADQFVDFMPKYMSYVKGIVDSDDLPLNVSREQLQQNKILKAISKRIVRKILDTFRKLYLDGKKNKDSLKEQLEKETDED---------------------------
PfHSP90_3         KQNSLKLYVRRVLVADEFVEFLPRYMSFVKGVVDSDDLPLNVSREQLQQNKILKAVSKRIVRKILDTFHKLYKEGKKNKETLRSELENETDEE---------------------------
                      :*:* *****:: : :*:*:*:  : *:****.:******* **:.*::* :.*::**.:* *:  :  .. .. .    ::   .  .

TaHSP90_3         KKESDKEAKD---------------KKSSWSSDLEKRKKKLEKKLKDVDKYTKFYNGFKGSLKVGCYDDDQNRKKIARLLRYKTLFSEK-ELTFDEYVEKMPEEQTEIYYVTSENYDDLK
TpHSP90_3         KNQRDKEENMEDNEKNKKG-----GKKSLWSADVELRRKGLEKRLKEVDRYSKFYNGFKGSLKVGCYDDDQNRKKIARLLRYKTLFSEK-ELTFDEYVDKMPEEQTEIYYVTSESYEDLK
ToHSP90_3         KKEAEKKKEEKKEEKKEKKEDNKEDKKEKWMEDMKLRKKKLERKLKEVDKYGKFYKGFKGSLKVGCYDDDQNRKKIARLLRYKTLFSEK-ELTFDQYVEAMKEEQTEIYYVTSENYEDLK
BeqHSP90_3        ------------------------------------KKKEIEKKIAQPTEFDKFYSSFKGALKVGCYDDDANRKKIAKLLKYKTSKHKDTEITLEQYIAEMPENQKDIYYASGDSYKEIK
BBovHSP90_3       ------------------------------------KKKELKTKISEKSTFDKFYQNFKGSLKVACYDDPANRKKIAKLLKYQTSKHKDEEITLEQYVAEMQEGQLLIYYASGESYDAIH
BBigHSP90_3       ------------------------------------KKTELTKKLTAKTTFDKFYQNFRGSLKVACYDDASNRKKIAKLLMYQTSKHKSDEITLDQYIAEMPETQKAIYYASGDSYNAIH
PVX_123745        ------------------------------------KKKEIQKKINEPSTYKLIYKEYRKYLKTGCYEDDINRSKIVKLLLFKTMLHPK-SISLDTYVENMKPDQKFIYYASGDSYEYLS
PCHAS_143930      ------------------------------------KKKEIQKKLNEPSVYKSIYKEYRKFFKSGCYEDDLNRSKIVKLLLFKTMHHPK-SISLDTYVENMKPDQKFIYYASGESYDYLS
PfHSP90_3         ------------------------------------KKKEITKKLSEPSTYKLIYKEYRKFLKSGCYEDDINRNKIAKLLLFKTMQYPK-SISLDTYIEHMKPDQKFIYYASGDSYEYLA
                                                      ::. :  ::     :  :*. ::  :* .**:*  **.**.:** ::*    . .:::: *:  *   *  ***.:.:.*. :

TaHSP90_3         QLPHIQGLKKRKYDVLYLHDTMDEGCLTKLEEHKGKKFKNVQKADLNLKLTDEEQKEEERKESKYKPLLLYLKDLLP-ETSGVKLSRRLVEDPCTVVASEWSMSSHMEKLMKSYAVHR-D
TpHSP90_3         QMPHLQGLKKRKFDVLYLHDTMDEGCLTKLEEHRGKKFKNVQKADLNLKLTDEEQKEEERKETKYKPLILYLKDLLP-ETSGVKLSRRLVEDPCTVVASEWSMSSHMEKLMKSYAVHR-D
ToHSP90_3         TMPHLQGLKKRKFDVLYLSDTMDESCLTKLEEYKGKKFKNVQKADLNLKLTEEEENEEKRKEAKYKPLLLYLKDMFP-ETSGVKLSRRLVEDPCTVVASEWSMSSHMEKLMKSYAVHR-D
BeqHSP90_3        NIPHLQGFVKRDLDVLFLMDTMDESCLTQLMDYEGKRFKSIQKGEISFDLTEEEKEEEKKKIKKYKPLTKVAKKMLP-ELLDVKVSRRLTDDPCTVVASEWGMTAHMEKLVKSYVVHKQE
BBovHSP90_3       NSPHLQGFRKRGIDVLYLTDTMDESCVTQMFEYEGRSFKSVQKGDVEFQRTEEDLEKDRVTMKKYAPLVKLFKQHID-DIYDVKLSHRLTDDPCTVVAADWGMSAHMEKIVKSYVVNKHE
BBigHSP90_3       SSPHLQGFHKRGIDVLYLTDTMDESCINQLYEYEGKSFKSVQKGDVDFERTQEEVERDELTLKKYAPLIKVLKENIS-EIYDVQLSRRLTDDPCIVVVTEWGMSPNMEKIVKSYVVNKKD
PVX_123745        KIPQLQIFKKKNIDVVFLTESVDESCVQRVQEYDGKKFKSIQKGEITFELTDDEKKKEEKVKKMYKALIDVISDTLRNQIFKVEISRRLVDAPCAVVSTEWGLSGQMEKLMKINVNNA--
PCHAS_143930      KIPQLQIFKKKNIDVLFLTESVDESCIQRVEDYDGKKFKSIQKGEITFDLTEEEKKREEKVKKMYTALIDVISDTLRNKIFKVEISRRLVDAPCAVVSTEWGLSGQMEKLMKININNT--
PfHSP90_3         KIPQLQIFKKKNIDVLFLTESVDESCIQRVQEYEGKKFKSIQKGEISFELTEEEKKKEQQMQKMYKALIDVISDTLKNQIFKVEISRRLVDAPCAVVSTEWGLSGQMEKLMKMNVSNS--
                    *::* : *:  **::* :::**.*: :: :: *: **.:**.:: :. *::: :.:.     * .*    .. :  .   *::*:**.: ** ** ::*.:: :***::*    :

TaHSP90_3         DSFDMFN-KLNSKVLELNPDHPIMVKLLYLYTQTQKLDKVEKKDEQKDKSVEKEEESKEDESKDKNEETKDETKSKEDESKDKETESKDEKELEEKRKELTEKKKKYMKRLDRLFKLLYN
TpHSP90_3         DSFDMFN-KLNSKVLELNPDHPIMVKLLYLYTQTQKLDQRN------DTERVEKERQQKDTEGEKDTEGEKDTEGEKDSEREEDKKPTDDQSTDVERRELLERKSRYMRRLDRLFKLLYN
ToHSP90_3         EKFETFN-KLSSKVLELNPDHPIMIKLLYLFTQAQNMLKEDKLDKE-DKEDKDKAAKLDKEDKHEDADKKKDESEEGAEKKESKDEDEEATAAVEQDPELVEKKKRYMKRLDRLFKLLYN
BeqHSP90_3        DSFDVAS-KFNSRILEINPDHPIMIELLKRAIKDPES-----------------------------------------------------------------------SDLKRSILLLYN
BBovHSP90_3       GDMNSYTSGLRSRILEINAEHPIMLEMLKRAIN-----------------------------------------------------------------------EADDSSFIDSIKLLYN
BBigHSP90_3       DDVDPFGSGMRSRILEINGDHPIMIELLKRCKN-----------------------------------------------------------------------DTVDSSVIDSMKLLYN
PVX_123745        ---DQIKAMSGQKILEINPDHPIMIDLLKRSVSNPKD-----------------------------------------------------------------------SQLTESIKIIYQ
PCHAS_143930      ---DHIKAMSGQKILEINPDHPIMIDLLKRSVENPKD-----------------------------------------------------------------------PELIESIKIIYQ
PfHSP90_3         ---DQIKAMSGQKILEINPNHPIMIDLLKRSVTNPKD-----------------------------------------------------------------------LELTNSIKIMYQ
                     :       .::**:* :****:.:*                                                                                  .   : ::*:
```

```
TaHSP90_3         AAKLKSGFVLEEPQLVVNYLYEKLNRSLGDFVEKDFEFTKNLTLDDFEVEKEEVKEPDGVENLEEQKKMDDEDVFKLDEVEYPGKEDE-------------------------KTPEERK
TpHSP90_3         AAKLKSGFVLEEPQLVVNYLYEKLNRSLGDFVERDFEFSKNLTLDDFEVDKVETPVPEHLKKMQLEMESTQSTQGFTQSTEGSTGSTEGSTGSTD-------TKSTQSTETESPEPKSPE
ToHSP90_3         AAKLKSGFVLEEPQLVVNYLYEKLNRSLGDVVERDYDKSKDLKLDDFEVPKVDVKVPEHVKRLQEQEEARKAAEASKGVALNLDAVDQKAEPVAEEEDEEEEVKKPKKKKEKTKTKEEEK
BeqHSP90_3        AAKLASGFIVENPKNVVQSAYKYLNHNLGVDASSKLEDVQVEESETEEEKPEDPGTLDIERLIESNN---------------------------------------------VEIDDEVK
BBovHSP90_3       AAKLAGGFTIENPSVISHSAYAYLSDKLKVDSSVTLEDIPYTPEPEKEDDPLKMPEGLELEEIDLDK-----------------------------------------------------
BBigHSP90_3       AAKLAGGFSIENPTTLTRTAYSYLSDQLNVDASATIDDIAYTP--EAEDETTESPEDMELEEIDLDD-----------------------------------------------------
PVX_123745        SAKLASGFDLEDTADLAQIVYDHINQKLGVDNNLKIDDLDPAIFETKKMEQEDSPDGQKFHEEINIDDEIQ-------------------------------------------------
PCHAS_143930      SAKLASGFDLEDTSDLAQIVYDHINQKLGVDNTLKIDDLDPAIFETKKIDDQN-------TEEINIDDAIK-------------------------------------------------
PfHSP90_3         SAKLASGFDLEDTADLAQIVYDHINQKLGVDNNLKIDDLDPSIFETKKIEDEN--DSSKFEEEINIDDEIQ-------------------------------------------------
                  :*** .** :*:.  : .  *  :. .*        :          :    .

TaHSP90_3         KQLEEASVDLKEILQGRGPEGFDLTGGTVGDTGGE-PMFDQFKSTGVDVDKLKSKEEDYDW--SNDEL
TpHSP90_3         EKGMDGGLELEEIFAGKDPEGFEVTRGSVDDAEGEQAMFDHLKSRGLDVEKLRSKQEDYDW--SNDEL
ToHSP90_3         KEFDEQFEAMNELFMGKDPEGYDVTRGEVDDLDESKMMLDTLKSRGLDVEKLRNKEEDYDW--ANDEL
BeqHSP90_3        ESLKTHLYESKHQTEGEPLNLDQFESLSDEEEEDDEDEDDDDHEPKTHEYDPKDSPVTEDI--ITDEL
BBovHSP90_3       ---------------------------------------------------------DGKP--ANDEL
BBigHSP90_3       ---------------------------------------------------------EGKP--VNDEL
PVX_123745        ----------------------------------------------------KQDAAPEAAP-KNDEL
PCHAS_143930      -----------------------------------------------------------KGDSKNDEL
PfHSP90_3         ---------------------------------------------------KKDNNVDNESNDKSDEL
                                                                                 ***
```
