# Supplementary figures and images for: Characterization of HSP90 isoforms in transformed bovine leukocytes infected with Theileria annulata
Source: Cell Microbiol. 2016 Oct 20;19(3):e12669. doi: 10.1111/cmi.12669 (PMC5333456; doi:10.1111/cmi.12669)

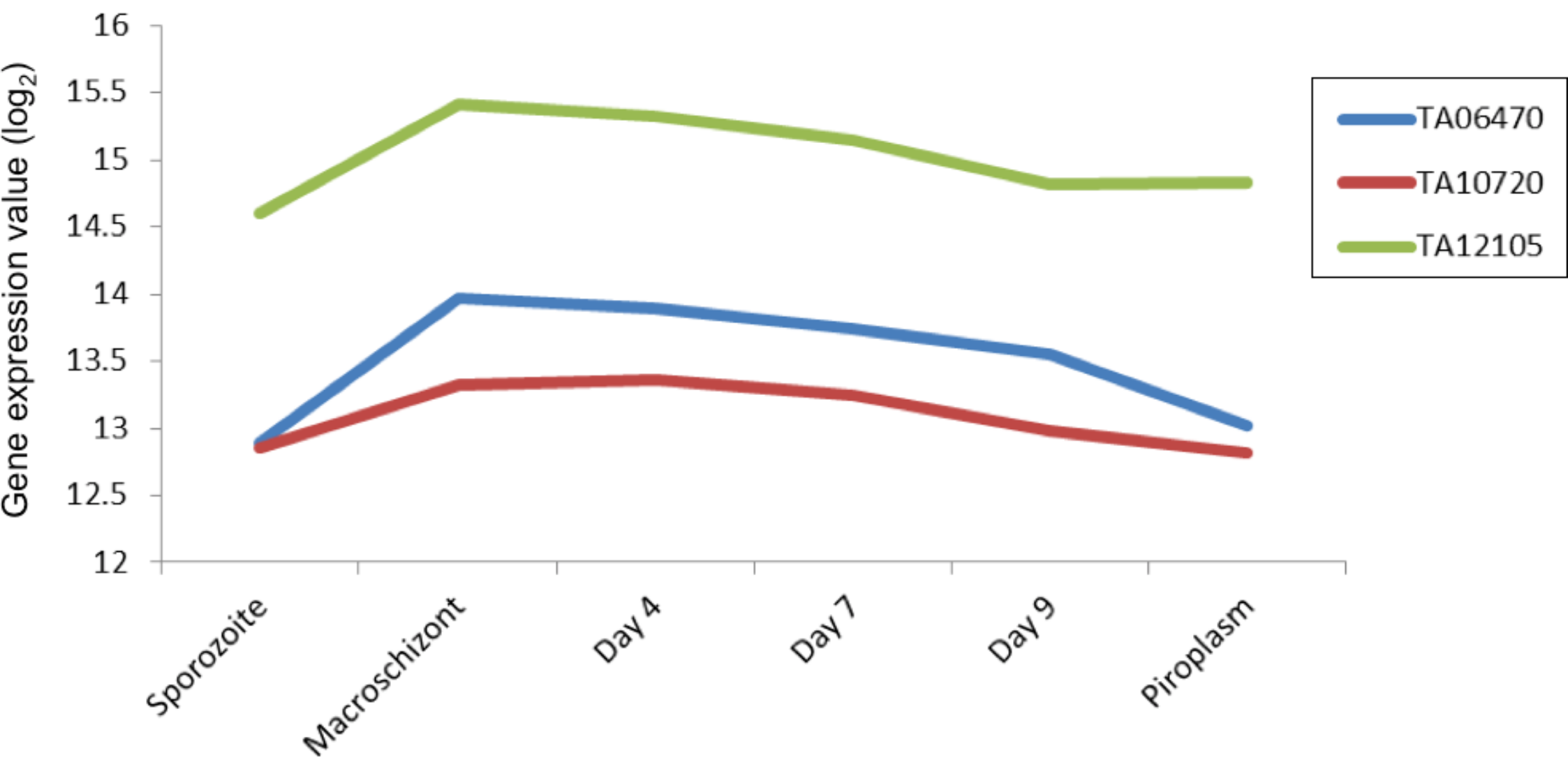

Supplement: Supplementary file 5 — Supporting info item [file CMI-19-na-s005.pdf]
