# Supplementary material for: Characterization of HSP90 isoforms in transformed bovine leukocytes infected with Theileria annulata
Source: Cell Microbiol. 2016 Oct 20;19(3):e12669. doi: 10.1111/cmi.12669 (PMC5333456; doi:10.1111/cmi.12669)
Supplement: Supplementary file 6 — Supporting info item [file CMI-19-na-s006.pdf]

Supplementary Table 1A. Summary of the different *T. annulata* HSP90 isoform properties

Locus	Predicted length (amino acid residues)	Predicted molecular weight (kDa)	Predicted Function
**TA12105**	722	83.8	Classical cytoplasmic HSP90
**TA06470**	988	115.6	ER form
**TA10720**	913	104.2	Apicoplast
**TA06845**	726	83.0	Similarity to Drosophila Trap

Supplementary Table 1B. NCBI Accession numbers for protein sequences used in this analysis

	NCBI Accession No
**Group 1 (TA12105 orthologues)**	
**TaHSP90_1**	XP_952473
**TpHSP90_1**	AAA30132.1
**BBovHSP90_1**	XP_001611554.1
**ToHSP_1**	XP_009690258
**BbigHSP90_1**	XP_012769365.1
**BeqHSP90_1**	XP_004828773.1
**PfHSP90_1**	XP_001348998.1
**PCHAS_HSP90_1**	XP_741381.2
**PVXHSP90_1**	XP_001613451.1
**TGME49_288**	XP_002368278.1
**CpHSP90_1**	XP_626924
**Group 2 (TA10720 orthologues)**	
**TaHSP90_2**	XP_953286.1
**TpHSP90_2**	XP_764281.1
**ToHSP90_2**	XP_009692206
**BbovHSP90-2**	XP_001611867.1
**BbigHSP90_2**	XP_012768918.1
**BeqHSP90_2**	XP_004830928.1
**PfHSP90_2**	XP_001348591.1
**PVXHSP90_2**	XP_001615952.1
**PCHASHSP90_2**	XP_737240.2
**TGME49_310**	XP_002364289.1
**Group 3 (TA06470 orthologues)**	
**TaHSP90_3**	XP_953842.1
**TpHSP90_3**	XP_766455.1
**ToHSP90_3**	XP_009689726.1
**BeqHSP90_3**	XP_004833528.1
**BBovHSP90_3**	XP_001610762.1
**BBigHSP90_3**	XP_012766459.1
**PVXHSP90_3**	XP_001617311.1
**PCHASHSP90_3**	XP_741379.2
**PfHSP90_3**	XP_001350620.1
**CpHSP90_3**	XP_628530
**TGME49_244**	XP_002366945.1
**Group 4 (TA06845)**	
**TaHSP90_4**	XP_954026.1
**TpHSP90_4**	XP_766274.1
**BBovHSP90_4**	XP_001611009.1
**BBigHSP90_4**	XP_012766750.1
**ToHSP90_4**	XP_009689549.1
**BeqHSP90_4**	XP_004833240.1
**PfHSP90_4**	XP_001347859.1
**PVXHSP90_4**	XP_001615300.1
**PCHASHSP90_4**	XP_743823.1
**TGME49_292**	XP_002370104.1
